# Supplementary figures and images for: Application of machine learning in in vitro propagation of endemic Lilium akkusianum R. Gämperle
Source: PLoS One. 2024 Jul 25;19(7):e0307823. doi: 10.1371/journal.pone.0307823 (PMC11271868; doi:10.1371/journal.pone.0307823)

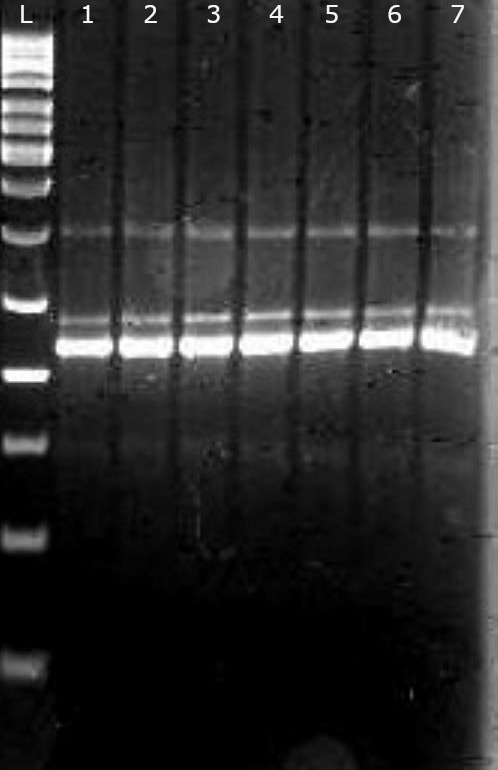

Supplement: S1 Fig — Raw images of Fig 1I given. DNA bands profile from left to right; fist is column DNA ladder; second column is mother plant and others belong to in vitro regenerated plants. (TIF) [file pone.0307823.s001.tif]
