# Supplementary material for: Application of machine learning in in vitro propagation of endemic Lilium akkusianum R. Gämperle
Source: PLoS One. 2024 Jul 25;19(7):e0307823. doi: 10.1371/journal.pone.0307823 (PMC11271868; doi:10.1371/journal.pone.0307823)
Supplement: S2 Fig — Raw data set belonging to shoot number, fresh shoot height and shoot weight from adventitious buds cultured on MS medium containing 1 mg/L NAA + 2 mg/L mT, 1 mg/L NAA + 2 mg/L BA and 0.5 mg/L NAA + 1 mg/L mT. (DOCX) [file pone.0307823.s002.docx]

**S2 Fig. Effects different medium on shoot number, fresh shoot height and shoot weight.**

| **Media** | **Shoot number** | **Shoot length** | **Shoot weight** |
| --- | --- | --- | --- |
| T6 | 2 | 7.5 | 0.6 |
| T6 | 1 | 7.8 | 0.81 |
| T6 | 3 | 7.2 | 0.52 |
| T6 | 2 | 7.5 | 0.59 |
| T6 | 4 | 9 | 1.86 |
| T6 | 5 | 9.4 | 1.92 |
| T6 | 3 | 8.6 | 1.8 |
| T6 | 4 | 9.1 | 1.86 |
| T6 | 3 | 8.6 | 0.41 |
| T6 | 5 | 8.9 | 0.48 |
| T6 | 2 | 8.3 | 0.33 |
| T6 | 2 | 8.5 | 0.42 |
| T6 | 2 | 8.1 | 1.6 |
| T6 | 1 | 8.5 | 1.3 |
| T6 | 3 | 7.8 | 1.5 |
| T6 | 2 | 8.2 | 1.9 |
| T6 | 4 | 7 | 1.22 |
| T6 | 6 | 7.4 | 1.35 |
| T6 | 2 | 7.1 | 1.17 |
| T6 | 5 | 6.5 | 1.13 |
| T6 | 3 | 9.5 | 1.24 |
| T6 | 5 | 9.1 | 1.28 |
| T6 | 4 | 9.8 | 1.21 |
| T6 | 3 | 9.6 | 1.23 |
| T6 | 3 | 8 | 2.96 |
| T6 | 4 | 7 | 2.83 |
| T6 | 4 | 9 | 3.09 |
| T6 | 2 | 8 | 2.94 |
| T6 | 2 | 10 | 0.33 |
| T6 | 1 | 12 | 0.26 |
| T6 | 2 | 7 | 0.38 |
| T6 | 3 | 11 | 0.35 |
| T6 | 3 | 7 | 1.53 |
| T6 | 2 | 5 | 1.49 |
| T6 | 3 | 9 | 1.63 |
| T6 | 4 | 7 | 1.47 |
| T10 | 3 | 9 | 0.6 |
| T10 | 5 | 7 | 0.64 |
| T10 | 4 | 11 | 0.58 |
| T10 | 1 | 9 | 0.62 |
| T10 | 4 | 13 | 1.86 |
| T10 | 5 | 11 | 1.79 |
| T10 | 5 | 16 | 1.87 |
| T10 | 2 | 12 | 1.91 |
| T10 | 4 | 6 | 1.4 |
| T10 | 3 | 8 | 1.37 |
| T10 | 3 | 5 | 1.43 |
| T10 | 4 | 6 | 1.44 |
| T10 | 3 | 13 | 1.6 |
| T10 | 5 | 12 | 1.68 |
| T10 | 2 | 14 | 1.54 |
| T10 | 4 | 11 | 1.61 |
| T10 | 5 | 14 | 1.22 |
| T10 | 5 | 15 | 1.25 |
| T10 | 3 | 14 | 1.19 |
| T10 | 4 | 13 | 1.23 |
| T10 | 3 | 15 | 2.96 |
| T10 | 4 | 16 | 3.01 |
| T10 | 5 | 16 | 2.87 |
| T10 | 3 | 14 | 2.98 |
| T10 | 4 | 6.5 | 0.33 |
| T10 | 5 | 7 | 0.31 |
| T10 | 2 | 8 | 0.35 |
| T10 | 4 | 5 | 0.34 |
| T10 | 5 | 11 | 1.53 |
| T10 | 4 | 9 | 1.57 |
| T10 | 6 | 10 | 1.49 |
| T10 | 4 | 13 | 1.55 |
| T10 | 3 | 8 | 0.9 |
| T10 | 3 | 7 | 1.05 |
| T10 | 1 | 8 | 0.89 |
| T10 | 5 | 8 | 0.98 |
| T12 | 4 | 9 | 1.1 |
| T12 | 5 | 8 | 1.2 |
| T12 | 5 | 11 | 1.18 |
| T12 | 3 | 9 | 1.08 |
| T12 | 3 | 10.5 | 1.2 |
| T12 | 3 | 11 | 1.31 |
| T12 | 2 | 9.5 | 1.28 |
| T12 | 3 | 8 | 1.12 |
| T12 | 4 | 7.4 | 1.3 |
| T12 | 5 | 8.5 | 1.42 |
| T12 | 5 | 8 | 1.28 |
| T12 | 4 | 7 | 1.33 |
| T12 | 3 | 13 | 1.1 |
| T12 | 4 | 11 | 0.98 |
| T12 | 3 | 11.5 | 1.25 |
| T12 | 4 | 15 | 1.13 |
| T12 | 3 | 14 | 1.7 |
| T12 | 3 | 12 | 1.62 |
| T12 | 3 | 15 | 1.69 |
| T12 | 4 | 14 | 1.82 |
| T12 | 4 | 12.3 | 2.2 |
| T12 | 5 | 13 | 1.87 |
| T12 | 3 | 11 | 1.93 |
| T12 | 4 | 14 | 2.12 |
| T12 | 6 | 7.5 | 1.8 |
| T12 | 4 | 9.5 | 1.85 |
| T12 | 7 | 7 | 1.78 |
| T12 | 5 | 7 | 1.74 |
| T12 | 4 | 11 | 1.2 |
| T12 | 5 | 9 | 1.25 |
| T12 | 3 | 13 | 1.18 |
| T12 | 5 | 11 | 1.2 |
| T12 | 5 | 8 | 1.4 |
| T12 | 7 | 8 | 1.49 |
| T12 | 3 | 6.5 | 1.35 |
| T12 | 5 | 9.5 | 1.38 |
