# Supplementary material for: Application of machine learning in in vitro propagation of endemic Lilium akkusianum R. Gämperle
Source: PLoS One. 2024 Jul 25;19(7):e0307823. doi: 10.1371/journal.pone.0307823 (PMC11271868; doi:10.1371/journal.pone.0307823)
Supplement: S1 Table — Raw data set belonging to callus induction rate and adventitious bud number per explants cultured on the MS medium supplemented with various concentrations of NAA and BA or mT. (DOCX) [file pone.0307823.s005.docx]

**S2 Table. Callus induction rate and adventitious bud number per explants.**

| **Medium** | **Callus formation (%)** | **Adventitious bud/explant** |
| --- | --- | --- |
| T0 | 0 | 0 |
| T0 | 0 | 0 |
| T0 | 0 | 0.16 |
| T0 | 0 | 0 |
| T0 | 0 | 0 |
| T0 | 0 | 0.33 |
| T0 | 0 | 0.5 |
| T1 | 16.6 | 0.83 |
| T1 | 33.3 | 1.5 |
| T1 | 60 | 0.66 |
| T1 | 16.6 | 1.83 |
| T1 | 16.6 | 0.33 |
| T1 | 83.3 | 0.66 |
| T1 | 50 | 1.5 |
| T2 | 33.3 | 0.66 |
| T2 | 16.6 | 0.83 |
| T2 | 50 | 0.66 |
| T2 | 66.6 | 0.5 |
| T2 | 50 | 0.33 |
| T2 | 75 | 0.33 |
| T2 | 33.3 | 0.83 |
| T3 | 75 | 0.83 |
| T3 | 50 | 0.66 |
| T3 | 50 | 0.33 |
| T3 | 75 | 0.5 |
| T3 | 66.6 | 0.5 |
| T3 | 100 | 0.33 |
| T3 | 100 | 0.66 |
| T4 | 0 | 2.16 |
| T4 | 0 | 1.5 |
| T4 | 16.6 | 2.16 |
| T4 | 33.3 | 2 |
| T4 | 0 | 0.83 |
| T4 | 16.6 | 1.16 |
| T4 | 33.3 | 0.66 |
| T5 | 0 | 0.83 |
| T5 | 0 | 0.66 |
| T5 | 33.3 | 1.5 |
| T5 | 83.3 | 1.83 |
| T5 | 0 | 1.5 |
| T5 | 33.3 | 0.33 |
| T5 | 33.3 | 1.83 |
| T6 | 16.6 | 2.16 |
| T6 | 33.3 | 2.33 |
| T6 | 33.3 | 3.16 |
| T6 | 50 | 2.5 |
| T6 | 50 | 3.16 |
| T6 | 75 | 1.83 |
| T6 | 75 | 3.16 |
| T7 | 16.6 | 1 |
| T7 | 33.3 | 0.6 |
| T7 | 60 | 0.83 |
| T7 | 0 | 1 |
| T7 | 16.6 | 1.5 |
| T7 | 83.3 | 0.83 |
| T7 | 0 | 1.8 |
| T8 | 33.3 | 1.16 |
| T8 | 50 | 1.5 |
| T8 | 33.3 | 2.16 |
| T8 | 66.6 | 2 |
| T8 | 50 | 1.5 |
| T8 | 50 | 2 |
| T8 | 66.6 | 1.16 |
| T9 | 75 | 1.16 |
| T9 | 100 | 1.5 |
| T9 | 83.3 | 2.16 |
| T9 | 66.6 | 2 |
| T9 | 83.3 | 1.5 |
| T9 | 100 | 2 |
| T9 | 75 | 1.16 |
| T10 | 16.6 | 4.58 |
| T10 | 0 | 4.91 |
| T10 | 16.6 | 3.5 |
| T10 | 0 | 5.66 |
| T10 | 16.6 | 6.58 |
| T10 | 0 | 4.83 |
| T10 | 16.6 | 4.83 |
| T11 | 12.5 | 2.16 |
| T11 | 16.6 | 1.5 |
| T11 | 16.6 | 1.83 |
| T11 | 33.3 | 2.16 |
| T11 | 16.6 | 2.33 |
| T11 | 16.6 | 1.66 |
| T11 | 33.3 | 1.5 |
| T12 | 66.6 | 2.16 |
| T12 | 33.3 | 3.66 |
| T12 | 75 | 3.66 |
| T12 | 50 | 2.5 |
| T12 | 33.3 | 3.16 |
| T12 | 50 | 2.5 |
| T12 | 50 | 3.16 |
