# Supplementary material for: Application of machine learning in in vitro propagation of endemic Lilium akkusianum R. Gämperle
Source: PLoS One. 2024 Jul 25;19(7):e0307823. doi: 10.1371/journal.pone.0307823 (PMC11271868; doi:10.1371/journal.pone.0307823)
Supplement: S2 Table — Raw data set belonging to root number, root length and survival rate of the plantlets. (DOCX) [file pone.0307823.s006.docx]

**S3 Table. Rooting behavior and survival rate of plantlets.**

| **Rooting Medium** | **Root Number** | **Root length** | **Survival Rate (%)** |
| --- | --- | --- | --- |
| R1 | 1 | 3.2 | 0 |
| R1 | 0 | 0 | - |
| R1 | 3 | 2.7 | 100 |
| R1 | 0 | 0 | - |
| R1 | 2 | 1.8 | 0 |
| R1 | 2 | 2.2 | 0 |
| R1 | 0 | 0 | - |
| R1 | 0 | 0 | - |
| R1 | 1 | 2.9 | - |
| R1 | 2 | 3.1 | 0 |
| R1 | 0 | 0 | - |
| R1 | 0 | 0 | - |
| R1 | 2 | 2.1 | 0 |
| R1 | 0 | 0 | - |
| R1 | 0 | 0 | - |
| R1 | 1 | 2.5 | - |
| R1 | 0 | 0 | - |
| R1 | 2 | 2 | 0 |
| R1 | 0 | 0 | - |
| R1 | 3 | 2.6 | 0 |
| R2 | 2 | 2.8 | - |
| R2 | 2 | 2.6 | - |
| R2 | 1 | 3.1 | - |
| R2 | 0 | 0 | - |
| R2 | 0 | 0 | - |
| R2 | 0 | 0 | - |
| R2 | 2 | 2.9 | 0 |
| R2 | 1 | 3.5 | - |
| R2 | 3 | 3.1 | 100 |
| R2 | 2 | 3.2 | - |
| R2 | 0 | 0 | - |
| R2 | 3 | 2.6 | - |
| R2 | 2 | 3.7 | 100 |
| R2 | 3 | 2.7 | 0 |
| R2 | 2 | 3.3 | 0 |
| R2 | 3 | 3.5 | 0 |
| R2 | 0 | 0 | - |
| R2 | 0 | 0 | - |
| R2 | 3 | 3.1 | 0 |
| R2 | 2 | 2.5 | 100 |
| R3 | 2 | 3.5 | 0 |
| R3 | 2 | 3.4 | 0 |
| R3 | 0 | 0 | - |
| R3 | 0 | 0 | - |
| R3 | 1 | 2.9 | - |
| R3 | 1 | 4.1 | 0 |
| R3 | 2 | 3.7 | 100 |
| R3 | 2 | 2.5 | - |
| R3 | 3 | 2.8 | - |
| R3 | 0 | 0 | - |
| R3 | 2 | 2.1 | - |
| R3 | 3 | 2.6 | - |
| R3 | 3 | 2.4 | 100 |
| R3 | 3 | 3.4 | 0 |
| R3 | 0 | 0 | - |
| R3 | 2 | 3.3 | - |
| R3 | 1 | 3 | - |
| R3 | 2 | 3.9 | 0 |
| R3 | 3 | 3.8 | 0 |
| R3 | 0 | 0 | - |
| R4 | 1 | 3.1 | - |
| R4 | 0 | 0 | - |
| R4 | 3 | 3.3 | 100 |
| R4 | 0 | 0 | - |
| R4 | 2 | 2.4 | 0 |
| R4 | 1 | 2.6 | - |
| R4 | 1 | 3.1 | - |
| R4 | 0 | 0 | - |
| R4 | 2 | 2.5 | 0 |
| R4 | 2 | 2.8 | 0 |
| R4 | 0 | 0 | - |
| R4 | 2 | 1.9 | - |
| R4 | 0 | 0 | - |
| R4 | 0 | 0 | - |
| R4 | 2 | 2.4 | 0 |
| R4 | 2 | 2 | 0 |
| R4 | 0 | 0 | - |
| R4 | 2 | 3.5 | 100 |
| R4 | 0 | 0 | - |
| R4 | 3 | 3.6 | 100 |
| R5 | 2 | 4.1 | - |
| R5 | 1 | 3.8 | - |
| R5 | 4 | 3.5 | - |
| R5 | 3 | 2.9 | - |
| R5 | 5 | 3.1 | - |
| R5 | 0 | 0 | - |
| R5 | 3 | 3.7 | - |
| R5 | 4 | 4.6 | 100 |
| R5 | 5 | 5.1 | 100 |
| R5 | 1 | 3.8 | - |
| R5 | 0 | 0 | - |
| R5 | 3 | 3.5 | - |
| R5 | 4 | 3.7 | 100 |
| R5 | 3 | 3.9 | 100 |
| R5 | 4 | 5.2 | 0 |
| R5 | 4 | 4.6 | 100 |
| R5 | 3 | 4.8 | 100 |
| R5 | 2 | 5.1 | - |
| R5 | 3 | 5.4 | 100 |
| R5 | 2 | 5.9 | - |
| R6 | 2 | 4.2 | - |
| R6 | 1 | 3.1 | - |
| R6 | 0 | 0 | - |
| R6 | 3 | 3.7 | 100 |
| R6 | 5 | 3.9 | 100 |
| R6 | 0 | 0 | - |
| R6 | 3 | 5.1 | 100 |
| R6 | 4 | 5.7 | 100 |
| R6 | 5 | 4.6 | 100 |
| R6 | 0 | 3.9 | - |
| R6 | 1 | 4.5 | - |
| R6 | 3 | 3.3 | - |
| R6 | 1 | 2.9 | - |
| R6 | 3 | 4.2 | 0 |
| R6 | 0 | 4 | - |
| R6 | 2 | 3.6 | - |
| R6 | 3 | 4.1 | - |
| R6 | 4 | 5.2 | 0 |
| R6 | 3 | 5.5 | 100 |
| R6 | 2 | 4.9 | - |
| R7 | 1 | 2.7 | 0 |
| R7 | 0 | 0 | - |
| R7 | 1 | 1.9 | 0 |
| R7 | 0 | 0 | - |
| R7 | 2 | 3.5 | 0 |
| R7 | 0 | 0 | - |
| R7 | 0 | 0 | - |
| R7 | 0 | 0 | - |
| R7 | 1 | 2.1 | 0 |
| R7 | 0 | 0 | - |
| R7 | 2 | 3.5 | 0 |
| R7 | 0 | 0 | - |
| R7 | 0 | 0 | - |
| R7 | 0 | 0 | - |
| R7 | 0 | 0 | - |
| R7 | 0 | 0 | - |
| R7 | 0 | 0 | - |
| R7 | 0 | 0 | - |
| R7 | 0 | 0 | - |
| R7 | 1 | 1.9 | 0 |
