# Supplementary material for: Application of machine learning in in vitro propagation of endemic Lilium akkusianum R. Gämperle
Source: PLoS One. 2024 Jul 25;19(7):e0307823. doi: 10.1371/journal.pone.0307823 (PMC11271868; doi:10.1371/journal.pone.0307823)
Supplement: Attachment — Submitted filename: PONE-D-24-00457_reviewer_1_.pdf [file pone.0307823.s008.pdf]

PLOS ONE

Integrating machine learning in endemic lily conservation: advanced micropropagation techniques for

Lilium akkusianum R. Gämperle

--Manuscript Draft--

**Manuscript Number:**	PONE-D-24-00457
**Article Type:**	Research Article
**Full Title:**	Integrating machine learning in endemic lily conservation: advanced micropropagation techniques for<br><br>Lilium akkusianum R. Gämperle
**Short Title:**	Advanced Micropropagation with Machine Learning for Lilium akkusianum
**Corresponding Author:**	Mehmet Tütüncü, Ph.D.<br>Ondokuz Mayıs Üniversitesi Ziraat Fakültesi: Ondokuz Mayis Universitesi Ziraat Fakultesi<br>ATAKUM, Samsun TURKEY
**Keywords:**	Endemic, Lilium; meta-topolin; adventitious bud; regeneration; Machine Learning
**Abstract:**	A successful regeneration protocol was developed for micropropagation of L. akkusianum R. Gämperle an endemic species to Türkiye from scale explants. The study also aimed to evaluate the effects of Meta-Topolin (mT) and N6-Benzyladenine (BA) on in vitro regeneration. The Murashige and Skoog medium (MS) supplemented with different levels of α-naphthaleneacetic acid (NAA)/BA and NAA/mT were used for culture initiation in the darkness. The highest callus rates were observed on explants cultured in MS medium with 2.0 mg/L NAA + 0.5 mg/L mT (83.31%), and the highest adventitious bud number per explant was 4.98 in MS medium with 0.5 mg/L NAA + 1.5 mg/L mT. Adventitious buds were excised and cultured in 16/8 h photoperiod conditions. The highest average shoot number per explant was 4.0 in MS medium with 2.0 mg/L mT + 1.0 mg/L NAA. Shoots were rooted with the highest rate (90%) in the medium with the 1.0 mg/L IBA, and the highest survival rate (87.5%) was recorded in rooted shoots in the same medium. The ISSR marker system showed that regenerated plantlets were genetically stable. Besides traditional tissue culture techniques used in current study, potential for improving the effectiveness of L. akkusianum propagation protocols by incorporating machine learning methodologies were evaluated. ML techniques enhance lily micropropagation by analyzing complex biological processes, merging with traditional methods. This collaborative approach validates current protocols, allowing ongoing improvements. Embracing machine learning in endemic L. akkusianum studies contributes to sustainable plant propagation, promoting conservation and responsible genetic resource utilization in agriculture.
**Order of Authors:**	Mehmet Tütüncü, Ph.D.
**Additional Information:**	
**Question**	**Response**
**Financial Disclosure**<br><br>Enter a financial disclosure statement that describes the sources of funding for the work included in this submission. Review the submission guidelines for detailed requirements. View published research articles from *PLOS ONE* for specific examples.	The author(s) received no specific funding for this work.

Powered by Editorial Manager® and ProduXion Manager® from Aries Systems Corporation

This statement is required for submission and **will appear in the published article** if the submission is accepted. Please make sure it is accurate.<br><br>**Funded studies**<br>Enter a statement with the following details:<br>• Initials of the authors who received each award<br>• Grant numbers awarded to each author<br>• The full name of each funder<br>• URL of each funder website<br>• Did the sponsors or funders play any role in the study design, data collection and analysis, decision to publish, or preparation of the manuscript?<br><br>Did you receive funding for this work?	
**Competing Interests**<br><br>Use the instructions below to enter a competing interest statement for this submission. On behalf of all authors, disclose any competing interests that could be perceived to bias this work—acknowledging all financial support and any other relevant financial or non-financial competing interests.<br><br>This statement is required for submission and **will appear in the published article** if the submission is accepted. Please make sure it is accurate and that any funding sources listed in your Funding Information later in the submission form are also declared in your Financial Disclosure statement.<br><br>View published research articles from *PLOS ONE* for specific examples.	The authors have declared that no competing interests exist.

**NO authors have competing interests**<br><br>Enter: *The authors have declared that no competing interests exist.*<br><br>**Authors with competing interests**<br><br>Enter competing interest details beginning with this statement:<br><br>*I have read the journal's policy and the authors of this manuscript have the following competing interests: [insert competing interests here]*<br><br>* typeset	
**Ethics Statement**<br><br>Enter an ethics statement for this submission. This statement is required if the study involved:<br><br>• Human participants<br>• Human specimens or tissue<br>• Vertebrate animals or cephalopods<br>• Vertebrate embryos or tissues<br>• Field research<br><br>Write "N/A" if the submission does not require an ethics statement.<br><br>General guidance is provided below. Consult the submission guidelines for detailed instructions. **Make sure that all information entered here is included in the Methods section of the manuscript.**	Ordu University, Akkuş Vocational School, Department of Forestry

**Format for specific study types**<br><br>**Human Subject Research (involving human participants and/or tissue)**<br>• Give the name of the institutional review board or ethics committee that approved the study<br>• Include the approval number and/or a statement indicating approval of this research<br>• Indicate the form of consent obtained (written/oral) or the reason that consent was not obtained (e.g. the data were analyzed anonymously)<br><br>**Animal Research (involving vertebrate animals, embryos or tissues)**<br>• Provide the name of the Institutional Animal Care and Use Committee (IACUC) or other relevant ethics board that reviewed the study protocol, and indicate whether they approved this research or granted a formal waiver of ethical approval<br>• Include an approval number if one was obtained<br>• If the study involved *non-human primates*, add *additional details* about animal welfare and steps taken to ameliorate suffering<br>• If anesthesia, euthanasia, or any kind of animal sacrifice is part of the study, include briefly which substances and/or methods were applied<br><br>**Field Research**<br><br>Include the following details if this study involves the collection of plant, animal, or other materials from a natural setting:<br>• Field permit number<br>• Name of the institution or relevant body that granted permission	
**Data Availability**<br><br>Authors are required to make all data underlying the findings described fully available, without restriction, and from the time of publication. PLOS allows rare exceptions to address legal and ethical concerns. See the PLOS Data Policy and FAQ for detailed information.	No - some restrictions will apply

A Data Availability Statement describing where the data can be found is required at submission. Your answers to this question constitute the Data Availability Statement and **will be published in the article**, if accepted.<br><br>**Important:** Stating 'data available on request from the author' is not sufficient. If your data are only available upon request, select 'No' for the first question and explain your exceptional situation in the text box.<br><br>Do the authors confirm that all data underlying the findings described in their manuscript are fully available without restriction?	
**Describe where the data may be found in full sentences. If you are copying our sample text, replace any instances of XXX with the appropriate details.**<br><br>• If the data are **held or will be held in a public repository**, include URLs, accession numbers or DOIs. If this information will only be available after acceptance, indicate this by ticking the box below. For example: *All XXX files are available from the XXX database (accession number(s) XXX, XXX.).*<br>• If the data are all contained **within the manuscript and/or Supporting Information files**, enter the following: *All relevant data are within the manuscript and its Supporting Information files.*<br>• If neither of these applies but you are able to provide **details of access elsewhere**, with or without limitations, please do so. For example:<br><br>*Data cannot be shared publicly because of [XXX]. Data are available from the XXX Institutional Data Access / Ethics Committee (contact via XXX) for researchers who meet the criteria for access to confidential data.*<br><br>*The data underlying the results presented in the study are available from (include the name of the third party*	All relevant data are within the manuscript and its Supporting Information files.

and contact information or URL).<br>• This text is appropriate if the data are owned by a third party and authors do not have permission to share the data.<br><br>* typeset	
Additional data availability information:	

Manuscript Click here to access/download;Manuscript;manuscript.docx

Integrating machine learning in endemic lily conservation: advanced micropropagation techniques for Lilium akkusianum R. Gämperle

Mehmet Tütüncü[1*]

[1]Department of Horticulture, Faculty of Agriculture, University of Ondokuz Mayıs, Samsun, Türkiye

*Corresponding author

E-mail: mehmet.tutuncu@omu.edu.tr (MT)

Abstract

A successful regeneration protocol was developed for micropropagation of *L. akkusianum* R. Gämperle an endemic species to Türkiye from scale explants. The study also aimed to evaluate the effects of Meta-Topolin (mT) and N6-Benzyladenine (BA) on in vitro regeneration. The Murashige and Skoog medium (MS) supplemented with different levels of α-naphthaleneacetic acid (NAA)/BA and NAA/mT were used for culture initiation in the darkness. The highest callus rates were observed on explants cultured in MS medium with 2.0 mg/L NAA + 0.5 mg/L mT (83.31%), and the highest adventitious bud number per explant was 4.98 in MS medium with 0.5 mg/L NAA + 1.5 mg/L mT. Adventitious buds were excised and cultured in 16/8 h photoperiod conditions. The highest average shoot number per explant was 4.0 in MS medium with 2.0 mg/L mT + 1.0 mg/L NAA. Shoots were rooted with the highest rate (90%) in the medium with the 1.0 mg/L IBA, and the highest survival rate (87.5%) was recorded in rooted shoots in the same medium. The ISSR marker system showed that regenerated plantlets were genetically stable. Besides traditional tissue culture techniques used in current study, potential for improving the effectiveness of *L. akkusianum* propagation protocols by incorporating machine learning methodologies were evaluated. ML techniques enhance lily micropropagation by analyzing complex biological processes, merging with traditional methods. This collaborative approach validates current protocols, allowing ongoing improvements. Embracing machine learning in endemic *L. akkusianum* studies contributes to sustainable plant propagation, promoting conservation and responsible genetic resource utilization in agriculture.

Keywords: Endemic, Lilium; meta-topolin; adventitious bud, regeneration, machine learning

Introduction

The members of the genus *Lilium* are perennial, herbaceous, and bulbous plants belonging to the Liliaceae family. More than 100 species represent the genus, mostly spread in temperate regions in the northern hemisphere [1]. It is well known that lilies have been used as ornamental plants in European gardens for centuries and decorative purposes since ancient times in Egypt and the Middle East [2]. *Lilium* species are still economically valuable ornamental plants with attractive and large flowers, which play an important role in the cut flower industry.

In Türkiye, the genus *Lilium* is represented by seven species (*Lilium akkusianum* R. Gämperle (endemic), *L. candidum* L., *L. ciliatum* P. H. Davis (endemic), *L. kesselringianum* Miscz., *L. martagon* L., *L. monadelphum* M. Bieb., and *L. ponticum* K. Koch.). *L. akkusianum* is one of the endemic *Lilium* species discovered by Ernst Gügel and Heinz-Werner Zaiss in 1993 and introduced to the scientific world by René Gämperle in 1998. It is distributed in a restricted area of the Akkuş district of Ordu province and on the border of Tokat province in the country [3]. *L. akkusianum* genotypes are naturally grown in moist deciduous forests at about 1200-1400 m. The species is often confused with *L. candidum* but is easily distinguished by some morphological features. The bulbs of L. akkusianum are similar to bulbs of *L. candidum*, but their color is whiter, and their scales are more lanceolate. The plant height of the genotypes is up to 1 m, the stem diameter is about 8-18 mm, and the number of flowers is between 1-14. The cream flowers are oval-elliptical and slightly curved back. Leaf margins and flower buds are woolly hairy. The plant becomes flowering in June and July, and flower life is longer [4]. Turkish lilies are mainly grown in the northern part of the country and are threatened by their restricted geographical ranges and small population sizes [5]. Additionally, *L. akkusianum* and other *Lilium* species are at risk of extinction due to urbanization in highlands and the destruction

of nature, overgrazing, unconscious over-harvesting of bulbs from nature and forest fire [6]. Therefore, urgent protection measures are needed for this valuable endemic plant.

In vitro regeneration techniques offer potential solutions for protecting threatened genetic resources [7] and lead to speedy large-scale commercial plant production and standardized, disease-free planting material [8]. Additionally, plant breeders and nurseries frequently employ in vitro techniques to accelerate the production of new clones with desirable traits. Although many parts of the plant may be used in the in vitro propagation of Lilium, in vitro scale culture is a superior propagation method for lilies [9]. However, in vitro regeneration from scale explants relies on many factors, such as cytokinin, auxin, and sucrose concentrations [10, 11]. Among the various plant growth regulators, the most efficient approach for establishing cultures and achieving in vitro mass propagation of lilies is carefully using appropriate concentrations of auxins and cytokinins [12]. Prior research findings have suggested that auxins 2,4-D (2,4-Dichlorophenoxyacetic acid), NAA (1-Naphthaleneacetic acid), IAA (Indole-3-acetic acid) and cytokinins Thidiazuron (TDZ) and BA (N6-Benzyladenine) are predominantly used as in the micropropagation of lilies viz., *L. pumilum* [13], Oriental Lilium Hybrid Cv. 'Ravenna' [14], *L. ledebourii* [15], *L. longiflorum* [16], *L. leucanthum* [17], *L. monodelphum* [18], *L. candidum* [19], *L. martagon* [20], and *L. longiflorum* [21].

Properly used cytokinins are crucial in regulating the cell cycle, developing plants and cell proliferation in micropropagation. However, exposing plants to inappropriate types or concentrations of cytokinins can adversely affect mass propagation [22]. In the past two decades, the application of meta-topolin (mT), originally extracted from poplar leaves, gained popularity in the field of in vitro plant propagation [23]. It has been indicated that difficulties like hyperhydricity, shoot-tip necrosis, poor rooting ability, and early senescence while creating effective micropropagation protocols can be alleviated using mT alternative to BA [24]. Additionally, positive effects of mT in micropropagation have been proven in shoot

regeneration of *Daphne mezereum* [25], *Gerbera jamesonii* [26], *Iris × hollandica* [27], *Tulipa* sp. L. [28], *Hypericum* sp. L. [29].

The tissue culture process encompasses various elements, such as the composition of the growth medium and environmental conditions, which can introduce genetic variations among the regenerated plants. Therefore, it is paramount to ensure the genetic consistency of these regenerated plants. This measure is critical to facilitate the successful proliferation and preservation of rare plant species [30]. In recent years, Polymerase Chain Reaction (PCR) techniques, specifically Inter-Simple Sequence Repeats (ISSR), have proven to be effective in assessing the genetic fidelity of regenerated plantlets in lilies [31, 32, 33].

Machine learning (ML) modeling has become popular in plant biotechnology, however there isn't much data to support it. This is especially true for in vitro plant tissue culture. Different machine learning algorithms have been used to optimize a variety of aspects, including in vitro sterilization [34], in vitro germination [35], in vitro cell culture [36], and in vitro somatic embryogenesis [34].

Similar to the human brain, artificial neural networks (ANNs) are composed of linked processing neurons that may interpret information via weighted communication links [37]. Artificial neural networks (ANNs) are used to empirically establish links between independent (input) and dependent (output) variables, making it easier to analyze the information exchange between the two.

One of the most prominent Artificial Neural Networks (ANNs) is the Multilayer Perceptron (MLP), which is organized into several levels, including an input layer, an output layer, and one or more hidden layers. Using both input and output variables as part of the training set, MLP uses a supervised training technique Eq.1.

XGBoost algorithm, a powerful tool for solving problems with classification and regression. It is well known for its exceptional performance and speed and is a member of the Gradient Boosting Decision Trees class. Within a gradient boosting architecture, XGBoost excels at learning from errors and progressively reducing the error rate over multiple iterations.

Successful shoot regeneration protocols have been reported for widely grown native *Lilium* species, but studies on mT-mediated in vitro regeneration in Lilium are very limited. Notably, no studies to date have reported establishing an efficient mass clonal propagation protocol for *L. akkusianum*, nor have they assessed the regenerated plants' genetic stability. Therefore, this study aimed to establish the in vitro regeneration potential of endemic *L. akkusianum* species from bulb-scale explants, comparing the effects of the commonly used BA with its new alternative mT in adventitious shoot regeneration. Additionally, to broaden the research scope, this study seeks to incorporate ~~Artificial Neural Network (~~AN[illegible]) analysis and machine learning methodologies. Employing computational techniques, the research aims to model and forecast the impacts of diverse culture media components and plant growth regulators on the quality of micropropagation.

Materials and methods

Plant material and surface sterilization

L. akkusianum bulbs were obtained from the genetic collection of Ordu University, Akkuş Vocational School, Department of Forestry after the flowering season in 2021. The bulbs were washed to remove soil, and roots were trimmed off from the bulbs. The excessive water was drained, and bulbs were stored at 4°C for two months before in vitro culture. After storage, the scales were washed under running tap water for two hours and pre-sterilized with $HgCl_2$ (0.1%) for 20 min. The scales were rewashed with sterile distilled water and transferred to sterile

conditions for further sterilizations. The scales were immersed in a 70% ethanol solution for 1-2 minutes and rinsed multiple times with sterile water to remove residual ethanol. They were then soaked in a 25% commercial bleach solution (Domestos®, 4.5% v/v) for 15 minutes, and the final step involved four additional washes with sterile distilled water. Following the surface sterilization process, the outer layers of the scales were carefully removed, leaving only the inner fleshy layer. This inner layer of the scale was then cut into small squares, approximately 0.5 cm x 0.5 cm, using a sterile scalpel. (Fig 1A).

Culture initiation and growth condition

The explants were cultured in Petri dishes (90 × 15 mm) containing 25 ml of full-strength MS medium [38] supplemented with 3% sucrose containing different concentrations and a combination of α-naphthaleneacetic acid (NAA) (0.5, 1.0, 2.0 mg/L) with 6-benzyl amino purine (BA) (0.5, 1.0, 2.0 mg/L) or Meta-Topolin (mT) (0.5, 1.0, 2.0 mg/L). Twelve different culture media, each with seven replicates (Petri dishes) and each replicate containing four explants were tested. The medium was solidified using 7 g/L agar, and the pH of the medium was adjusted to 5.6–5.8 before autoclaving at 121 °C and 15 psi for 15 min. All explants were cultured at 23± 2 °C in constant darkness for 2 months and subcultured every 4 weeks.

After the first subculture, the explants started producing adventitious bud or callus. They were transferred to a fresh culture medium for further growth and development. The experiment for regeneration induction from scale was repeated twice, and then callus rate (%) and adventitious bud per explant were recorded at the end of each experiment. Histological studies were conducted by sampling calli with adventitious bud to track bud development stages.

Effects of different mT and BA on shoot proliferation

Adventitious buds were excised and cultured in the jar (0.6 L) (Fig 1E) containing MS medium containing 1 mg/L NAA + 2 mg/L mT, 1 mg/L NAA + 2 mg/L BA and 0.5 mg/L NAA + 1 mg/L mT which exhibited the highest number of adventitious bud per explant during initiation stage. Each culture medium has nine replicates (jars). The cultures were maintained in a growth room at a temperature of 23 ± 2 °C, with a light/dark cycle of 16 hours of light and 8 hours of darkness. Cool white fluorescent lamps provided illumination with a photosynthetic photon flux density (PPFD) of 70 µmol m□^{2}s□^{1}. The explants were cultured for 3 months in the same medium, and shoot height (cm), shoot fresh weight (g) and shoot number per explant was observed.

In vitro rooting and acclimatization

Mature shoots of *L. akkusianum* were carefully cut from the clusters of shoots (Fig 1F) and then transplanted into jars (0.6 L) containing 50 ml MS medium with varying concentrations of NAA or IBA (0.5, 1.0, 2.0, 3.0 mg L□^{1}) to induce root development. In total, seven different rooting media with five replicates and each replicate has four explants, were used. All cultures were kept in the growth room under the same conditions with a shoot proliferation stage for six weeks. Then, rooting rate, root number per explant, and root length were measured.

Plantlets were picked up from the culture medium, and residues of the culture medium were removed by washing them under running tap water (Fig 1H). After removing residues, plants were dipped in fungicide solution (Captan 50WP) at 2.5 g L-1 for 10-15 s and transferred to a grow box with a plastic cover containing sterile peat-perlite substrate (1:1 v/v). The plants were kept in the grow box (Fig 1H) in the growth room at 23± 2 °C under a 16 h photoperiod and 70 µmol m□^{2}s□^{1} cool white fluorescent lamps for 2 weeks and then transferred to greenhouse conditions, and the plastic cover was gradually removed. After adaptation to greenhouse

conditions, the plantlets were transferred to plastic pots (0.9 L) (Fig 1H) containing peat-perlite substrate (1:1 v/v) and irrigated twice weekly with 200 ml water per pot. No chemicals were used during cultivation for pests and diseases.

Histological analysis

Callus with adventitious buds was randomly selected from the different culture mediums and fixed in FPA-70 (formaldehyde□propionic acid□alcohol) solution until examined [39]. All fixed samples were dehydrated in ethanol and tert-butanol series and embedded into paraffin. Paraffin-embedded samples were sectioned longitudinally (10μ) with a rotary microtome (RM2134, Leica, Wetzlar, Germany), stained with 0.125% hematoxyline buffered with KMnO4 and mounted in Entellan [40]. Preparations were observed with a fluorescence-light microscope (Olympus BX51, Tokyo, Japan) and visualized by a digital micrograph system (Olympus DP72, Tokyo, Japan).

Fig 1. In vitro regeneration in L. akkusianum. (A) Explant preparation. (B) Adventitious bud regeneration in M0 medium 5-6 weeks of culture in the dark. (C) Callus and adventitious buds regeneration in M9 medium 5-6 weeks of culture. (D) Callus and adventitious buds regeneration in M10 medium 5-6 weeks of culture. (E) Shoot development in M12 medium 2 weeks after culture initiation under 16/8 h photoperiod. (F) Later shoots development and proliferation under 16/8 h photoperiod (G) Plantlets with well-developed roots. (H) Acclimatized plantlets in the grow box (I) Plantlets 2 months after transfer into the peat-perlite substrate (1:1 v/v). Scale bars: B, D, E, F = 10 mm; A, C, G, H = 1 cm; I =5 cm.

Genetic stability of in vitro-grown plantlets

The genetic stability of the in vitro-grown plantlets was evaluated using the ISSR marker system. Leaves from six in vitro-grown plantlets and a mother plant (totally seven genotypes) were collected and frozen in liquid nitrogen. DNA isolation was performed based on a protocol for minipreps described by Edwards et al. [41] The quality and quantity of genomic DNA were evaluated spectrophotometrically (NanoDrop ND 100, NanoDrop Technologies). Fourteen ISSR primers (Table 1) were used to assess genetic stability [42]. Amplification was carried out in a volume of 25 µL (10 ng of L. akkusianum DNA template, 2X PCR Mastermix (Fermentas K0171, Waltham, MA, USA), 25 mM MgCl2, 10 µM primer, and 1 U of Taq DNA polymerase (Fermentas EP0402)) according to following program in a Veriti thermal cycler (Applied Biosystems): initial denaturation at 94 °C/3 min, 35 cycles of 94 °C/45 sec, 55 °C/1 min, 72 °C/45 sec, and final elongation at 72 °C/7 min. PCR products were then held at 4 °C.

The amplified products were separated on 1.5% agarose gel in 0.5X TAE buffer (40 mM Tris-acetate, 1 mM EDTA, pH 8.0) for 3.5 h at 80 V. The gels were stained with 0.5 g/mL ethidium bromide. A 1 kb DNA ladder (Fermentas) was used to determine the fragment size. Fragment patterns were photographed by a gel documentation system (Genesys) under UV light. DNA band profiles resulting in ISSR analysis were visually checked. It was determined whether there were any differences between the control sample and those obtained from tissue culture studies.

Table 1. ISSR primer sequence [42].

No	Primer code	Sequence (5’-3’)
1	UBC807	AGA GAG AGA GAG AGA GT
2	UBC808	AGA GAG AGA GAG AGA GC
3	UBC810	GAG AGA GAG AGA GAG AT
4	UBC811	GAG AGA GAG AGA GAG AC
5	UBC812	GAG AGA GAG AGA GAG AC
6	UBC815	CTC TCT CTC TCT CTC TG
7	UBC816	CAC ACA CAC ACA CAC AT
8	UBC818	CAC ACA CAC ACA CAC AG
9	UBC820	GTG TGT GTG TGT GTG TC
10	UBC827	ACA CAC ACA CAC ACA CG
11	UBC834	AGA GAG AGA GAG AGA GT

12	UBC835	AGA GAG AGA GAG AGA GC
13	UBC845	CTC TCT CTC TCT CTC TG
14	UBC850	GTG TGT GTG TGT GTG TC

Experimental design and statistical analysis

Experiments were established based on a completely randomized design. Means of all data were separated by variance analysis, and significant differences ($p < 0.05$) were evaluated with LSD test using the JMP® program (SAS Institute, Cary, NC). The percentage values were arc-sin transformed prior to the variance analysis.

XGBoost and MLP analysis

Extreme Gradient Boosting (XGBoost) and Multilayer Perceptron (MLP) were used in this study to model and predict adventitious bud induction, shoot proliferation, and rooting from scale explants of *L. akkusianum*. The dataset was split into training and testing subsets using a five-fold cross-validation technique in order to thoroughly evaluate the MLP and XGBoost's predictive performance.

The target (output) variables included adventitious buds, shoot length, number of shoots, shot weight, root length and number of rooting plants, and number of roots and root length. The input variables included rooting and shooting medium. Caret and Kernlab packages were utilized to facilitate the coding process using the R programming language.

Metrics such as the coefficient of determination (R2), which calculates the strength of the relationship between the model and dependent variable, Root Mean Square Error (RMSE), which indicates how closely the regression line matches the observed data points, and Mean Absolute Error (MAE), which calculates the average amount of error between the observed and

predicted values, were used to assess and compare the efficacy and precision of the models. Eqs 1-3.

$$R^2 = 1 - \frac{\sum_{i=1}^{n}(Y_i - \hat{Y}_i)^2}{\sum_{i=1}^{n}(Y_i - \tilde{Y})^2} \quad (1)$$

$$RMSE = \sqrt{\frac{\left(\sum_{i=1}^{n}(Y_i - \hat{Y}_i)^2\right)}{n}} \quad (2)$$

$$MAE = \frac{1}{n}\sum_{i=1}^{n} |Y_i - \hat{Y}_i| \quad (3)$$

While Y_i = actual value, $\hat{Y}_i$ = Predicted value, $\tilde{Y}$ = mean o the actual values and n = sample count.

Results

Regeneration capacity of scale explants

The scale explants of *L. akkusianum* were cultured on the MS medium containing auxin and cytokinin combinations to induce in vitro regeneration (Fig 1A). In the fourth week of the culture, explants began to swell, and yellowish and friable callus tissues formed after the four to six weeks of the culture. At this stage, adventitious buds developed directly from explants or callus structures (Fig 1B, C, D). Variance analysis of callus induction rate (%) and adventitious bud number per explant has shown that the effects of plant growth regulators (PGRs) were

statistically significant (Table 2). Compared with control treatments (MS medium without PGRs), culture medium supplemented with PGRs induced callus formation.

The highest callus rates were observed on explants cultured on MS medium supplemented with 2.0 mg/L NAA + 0.5 mg/L mT (83.31%) and MS medium with 2.0 mg/L NAA + 0.5 mg/L BA (73.8%) while callus induction rate in the MS medium without PGRs (M0) was the lowest. After six weeks of the culture initiation, adventitious bud regeneration was observed on all culture mediums. The addition of PGRs to the medium increased the average number of adventitious bud formation per explant. The highest adventitious bud number per explant was 4.98 in M10 treatments (MS + 0.5 mg/L NAA + 1.5 mg/L mT), and the lowest was 0.14 in M0 (control).

Table 2. Callus induction rate and adventitious bud number per explants cultured on the MS medium supplemented with various concentrations of NAA and BA or mT.

Medium No	NAA (mg/L)	BA (mg/L)	mT (mg/L)	Callus (%) ± SE*	Adventitious bud/explant ± SE **
M0	-	-	-	0.0 ± 0.0 g (3.55)	0.14 ± 0.07 g
M1	0.5	0.5	-	39.48 ± 9.1 b-e (38.43)	1.04 ± 0.19 ef
M2	1.0	0.5	-	46.4 ± 7.1 bcd (42.74)	0.59 ± 0.07 fg
M3	2.0	0.5	-	73.8 ± 7.2 a (63.52)	0.54 ± 0.06 fg
M4	0.5	1.0	-	14.25 ± 5.2 fg (16.93)	1.49 ±0.22 cde
M5	1.0	1.0	-	26.17 ± 10.5 def (24.51)	1.21 ±0.20 de
M6	1.0	2.0	-	47.6 ± 7.6 bc (43.50)	2.61 ±0.19 b
M7	0.5	-	0.5	29.97 ± 10.9 c-f (28.56)	1.08 ±0.15 ef
M8	1.0	-	0.5	49.97 ± 4.7 bc (44.98)	1.64 ± 0.14 cd
M9	2.0	-	0.5	83.31 ± 4.4 a (69.49)	1.64 ± 0.14 cd
M10	0.5	-	1.0	9.48 ± 3.1 fg (13.73)	4.98 ± 0.33 a
M11	1.0	-	1.0	20.78 ± 3.0 ef (26.76)	1.87 ± 0.11 c
M12	1.0		2.0	51.17 ± 5.4 b (45.74)	2.97 ± 0.20 a

*LSDcallus = 6.23, LSDadv.bud=0.414, Levels not connected by the same letter are significantly different ($p < 0.05$), SE: Standard error.

The effects of different cytokinin on shoot proliferation

Adventitious buds were excised and cultured on the same medium individually at 23± 2 °C under 16/8 h light/dark photoperiod to assess their effects on shoot development (Fig 1E). There were no statistically significant effects of culture mediums on shoot weight and height. However, the effects of culture mediums on average shoot number per explant were statistically significant (Fig 2). Average fresh shoot weight varied from 1.3 g to 1.42 g in MS medium supplemented with 2.0 mg/L NAA + 1.0 mg/L BA (M6) and 2.0 mg/L NAA + 1.0 mg/L mT (M12) respectively. The highest average shoot height was 10.6 cm observed in the MS medium supplemented with 0.5 mg/L NAA + 1.0 mg/L mT, while the lowest was in the M6 medium with 8.3 cm. The effects of treatments on shoot number were statistically significant. The highest average shoot number per explant ranged from 3.7 to 4.0 in M10 and M12 medium, showing that MS medium supplemented with mT instead of BA was a superior culture medium for shoot proliferation.

Fig 2. Effects of plant growth regulator combination on shoot number, fresh shoot height and shoot weight in the sixth week of culture (*$p < 0.05$, $LSD_{shootnumber} = 0.876$; Levels not connected by the same letter are significantly different).

In vitro rooting and acclimatization of plantlets

Effects of NAA and IBA on average rooting percentage, root number per explant, root length and survival rate of the plantlets were statistically significant (Table 3). In comparison with the control group, adding auxin into the medium increased the rooting capacity of the explants. The shoots cultured on MS medium supplemented with 1.0 mg/L and 2.0 mg/L NAA or IBA had a superior rooting rate, root number, root length and survival rate than other treatments. However,

the highest rooting percentage (90%), the average root number per explant (2.8), and the average root length per explant (3.83 cm) were recorded in the treatment of MS medium supplemented with 1.0 mg/L IBA (R5 medium). The lowest values for the parameters were observed in the R7 medium (MS medium without PGRs). Rooted plantlets were hardened in a grow box containing peat-perlite substrate (1:1 v/v), and the survival rates were recorded two weeks later. The highest survival rate %) was 87.5 and 62.5% in R5 and R6 medium, respectively. There were no living plantlets in the control group two weeks after hardening. Overall, the results of the rooting experiment showed that IBA treatments are better than NAA.

Table 3. Effects of different NAA and IBA on rooting behavior and survival rate of plantlets.

Rooting Medium	NAA (mg/L)	IBA (mg/L)	Rooting (%)*	Root number per explant**	Root length per explant***	Survival rate (%)****
R1	0.5	-	50.0 ± 11.3 cd (45.0)	0.95 ± 0.23 cd	1.25 ± 0.28 cd	12.5 c (11.25)
R2	1.0	-	70.0 ± 10.2 abc (63.0)	1.55 ± 0.25 bc	2.13 ± 0.31 bc	37.5 bc (33.75)
R3	2.0	-	75.0 ± 9.6 abc (67.5)	1.6 ± 0.24 bc	2.37 ± 0.32 b	25.0 bc (22.50)
R4	-	0.5	60.0 ± 10.9 bc (54.0)	1.15 ± 0.23 cd	1.66 ± 0.31 bcd	37.5 bc (33.75)
R5	-	1.0	90.0 ± 6.7 a (81.0)	2.8 ± 0.32 a	3.83 ± 0.33 a	87.5 a (78.75)
R6	-	2.0	80.0 ± 8.9 ab (72.0)	2.25 ± 0.35 ab	3.81 ± 0.33 a	62.5 ab (56.25)
R7	-	-	30.0 ± 10.2 d (27.0)	0.4 ± 0.14 d	0.78 ± 0.27 d	-

~~Arc-sin transformed values are indicated in the parentheses,~~ Levels not connected by the same letter are significantly different ($p < 0.05$). *LSDrooting = 23.39, **LSDrootnumber = 0.70 ***LSDrootlength = 0.83 ****LSDsurvivalrate = 26.16.

Histological analysis of adventitious bud regeneration

Paraffin-embedded samples from the culture initiation experiment showed the formation of new cells on the initially developing calli, and the initiation of cell divisions started at 2-4 weeks of the culture (Fig 3A, B). The examinations showed that the cell divisions increased and formed the early stage of adventitious bud (Fig 3C, D, E). In addition, these types of structures were abundant on the explants cultured on MS medium supplemented with NAA and mT at the 4-6

weeks of the culture. In the final stage of adventitious bud development, they became bipolar plantlets with shoot apical meristem.

Fig 3. Histological observations on developmental stages of plant regeneration and genetic stability of in vitro regenerated plants. (A, B) General view of callus (C) and new cells. (C) Cell division and morphogenesis (D, E) Initiation of adventitious bud on callus tissue (black arrow). (E) Mature adventitious buds with shoot apical meristem (SA) and cotyledone tissues (Co), (G) DNA bands profile of in vitro regenerated plantlets (2-7) and mother plant (1). Scale bars: A, B, D, E and F: 100 µm, C: 20 µm

Genetic stability of the regenerated plantlets

The genetic stability of in vitro adventitious bud-derived plantlets was tested using ISSR markers. According to agarose gel electrophoresis results, DNA profiles of regenerated plantlets and mother plants did not differ. An ISSR gel image is presented in Fig 3G.

XGBoost and MLP models

The XGBoost and MLP algorithms were used to analyze the data obtained about the adventitious induction of buds and the proliferation of shoots from scale explants of L. akkusianum. The outcomes were validated and predicted using three distinct performance metrics. The MLP model yielded results with R2 values ranging from 0.13 to 0.83. Shoot Weight had the lowest R2 (r2 = 0.24), whereas Root Length had the highest (r2 = 0.83). The shoot weight had the lowest Mean Absolute Error (MAE) value (0.45), with low values frequently displayed. Root Mean Square Error (RMSE) values ranged from 0.60 to 2.74, with shoot weight and shoot length having the lowest and highest RMSE values, respectively Tabel 3. Regarding the XGBoost model, the root length had the highest R2 (r2 = 0.87) and the shoot

weight had the lowest (r2 = 0.28), with R2 values ranging from 0.28 to 0.87. Overall, the MAE values ranged from 0.40 to 0.87. The shoot length showed the highest RMSE value, and the adventitious bud showed the lowest RMSE (Table 4). The distribution of predited and actual values are presented in Figs 4-5. The results provided suggest that, when compared to their relative performances, XGBoost appears to perform better than the MLP model. It demonstrated competitive RMSE outcomes, lower MAE, greater R2 values, and remarkable performance in predicting Root Length across all evaluated plant characteristics.

Table 4. Performance Metrices of the MLP and XGBoost Models

	**Model**	**RMSE**	**R^2**	**MAE**
Adventitious Bud	MLP	0.61	0.74	0.51
	XGBoost	0.52	0.84	0.42
Root number	MLP	0.81	0.70	0.53
	XGBoost	0.79	0.69	0.52
Root Length	MLP	0.73	0.83	0.51
	XGBoost	0.65	0.87	0.44
Shoot number	MLP	1.33	0.18	1.07
	XGBoost	1.05	0.32	0.87
Shoot length	MLP	2.74	0.35	1.81
	XGBoost	1.21	0.80	0.88
Shoot weight	MLP	0.60	0.13	0.45
	XGBoost	0.54	0.28	0.40

MLP: Multilayer perceptron; RBF: Radial basis function; XGBOOST: Extreme Gradient Boost, R2: Coefficient of determination; MAE: Mean absolute error; RMSE: Root mean square.

Fig 4. Actual against predicted values of (A) adventitious bud, (B) number of roots, (C) root length, (D) number of shoots, (E) shoot length (F) shoot weight using MLP model analysis.

Fig 5. Actual against predicted values of (A) adventitious bud, (B) number of roots, (C) root length, (D) number of shoots, (E) shoot length (F) shoot weight using XGBoost model analysis.

Discussion

This study focused on the in vitro regeneration of *L. akkusianum* and the effects of mT on micropropagation in lilies for the first time. The highest callus rates were 73.8-83.31% observed in media containing 2.0 mg/L NAA + 0.5 mg/L BA and 2.0 mg/L NAA + 0.5 mg/L mT, respectively. The highest adventitious bud formation per explant was 4.98, obtained from MS medium supplemented with 0.5 mg/L NAA + 1.0 mg/L mT. The results have shown that successful callus and adventitious bud regeneration obtained and MS medium supplemented with NAA and mT instead of BA produced better results.

In previous studies, various types of auxin and cytokinin combinations and concentrations were successfully used in the in vitro regeneration of the *Lilium* species. Bakhshaie et al. [43] reported the highest percentage of calluses (65.55%) obtained from scale explants cultured on MS medium supplemented with 0.54 µM NAA and 0.44 µM BA in *L. ledebourii*. Fu et al. [44] observed that MS with 1.0 mg/L NAA and 0.5 mg/L BA were appropriate for inducing callus (64.67%) in L. brownii var. viridulum. Tang et al. [17] suggested that MS medium containing 0.5 mg/L BA and 1.0–3.0 mg/L 2,4-D was the best for callus induction from scales with the highest percentage (98.3%). Additionally, Nam and Kim [45] indicated that the most successful initiation of adventitious buds from scale explants occurred under dark conditions when placed on MS medium enriched with 0.5 mg/L BAP and 0.1 mg/L NAA. Conversely, the most effective callus induction was observed when microscales were incubated in complete darkness for 8 weeks on MS medium containing 1.0 mg/L 2,4-D and 0.1 mg/L BAP. These results show that the callus induction rate varied mainly based on the medium, species, type, and auxin and cytokinin balance and in agreement with the current findings in *L. akkusianum* that a higher level of auxin than cytokinin concentration in the medium led to callus induction. In contrast, higher cytokinin than auxin resulted in adventitious bud induction. Additionally, darkness one

of the major factors to induce callus formation, because culture initiation in complete darkness can lead to callogenesis due to increased endogenous IAA levels [46]. Therefore, higher cytokinin than auxin in the medium combining with light conditions and may be preferred to induce direct regeneration, while higher auxin than cytokinin in the medium and complete darkness for callogenesis and indirect regeneration for *L. akkusianum*.

Adventitious buds of *L. akkusianum* were cultured on MS medium supplemented with the combination of NAA, mT and BA. The highest shoot proliferation was obtained from the medium with mT and NAA. However, the effects of the mT or BA combined with NAA in the medium on shoot weight and shoot length were not statistically significant. Similar studies were conducted in ornamentally valuable bulbous plants to compare the effects of different auxin and cytokinin combinations on in vitro shoot proliferation. Sochacki et al. [28] reported that MS medium with 0.1 mg/L 2iP + 0.1 mg/L NAA + 5 mg/L mT produced the highest shoot number with 9.14 per explant in tulip. In another study in *Iris* × *hollandica* Tub. cv. professor Blaauw, MS + 1.0 mg/L mT + 0.25 mg/L NAA resulted in a considerably higher shoot number per explant with 17.53 and a mean shoot length with 7.06 cm [27]. In agreement with previous studies, MS supplemented with 2.50 mg/L mT with 0.1 mg/L NAA enhanced the formation of healthy and uniformly grown shoots per node and better shoot length in *Withania coagulans* (Stocks) Dunal [47]. In in vitro scale culture of *Cyrtanthus contractus*, a medicinal and ornamental plant, MS medium supplemented with 1-10 µM mT increased the number of shoots per explant, but fresh and dry mass of the shoots decreased in higher concentrations. These results are consistent with the present study for mT.

In vitro rooting and acclimatization of in vitro grown plantlets are crucial steps for optimization regeneration protocol for endemic and under-threatened plants. In the current study, the effects of different concentrations of NAA and IBA on rooting behavior in *L. akkusianum* were investigated. Adding IBA into the medium was superior to NAA for rooting rate, root number,

and root length. Moreover, rooted plantlets in the medium with IBA have prolonged survival rate. Superior effects on rooting of in vitro grown plantlets in *Lilium* species were also indicated previously. In accordance with the current study, Rafiq et al. [14] compared IBA and NAA performance in rooting of in vitro regenerated shoots from scale explants of Oriental Lilium Hybrid cv. 'Ravenna'. They reported the highest rooting rate of 92.71%, primary root number per shoot of 12.06, root length of 3.17 cm and survival rate of 98.96% obtained from MS medium supplemented with 1.5 mg/L IBA. Liu and Yang [48] used a half-strength MS medium supplemented with four different IBA concentrations from 0.49 to 4.9 μM for rooting in vitro regenerated shoots of *L. orientalis*. They observed that as the concentration of IBA in the medium increases, the mean root number, mean root length and rooting rate also increase. Additionally, medium supplemented with IBA was found the most favorable results for rooting activity in *L. longiflorum* [21, 49] and *L. napalense* [50]. In contrast, Yang et al. [33] employed IBA and NAA for in vitro rooting of shoots in *Lilium davidii* var. *unicolour* Salisb. They discovered that all culture media supplemented with different NAA and IBA resulted in root formation (85-100%). However, NAA was more effective on root number and root length. The results of previous studies and the present study may suggest that rooting response to auxin type and concentrations could related to explants, species and culture conditions. Additionally, a developed root system increases the survival rates of the in vitro grown plantlets.

Histological analysis is key in illustrating developmental pathways of in vitro regeneration. In the present study, the formation of callus tissues initiated from the epidermal layer and eventually developed adventitious buds. Callus formation is not desired in organogenesis due to the potential for somatic variations to arise in the shoots regenerated from callus tissue [32]. However, the genetic stability of the regenerated plantlets was assessed using the ISSR marker with 14 primers in *L. akkusianum*; no polymorphic band was detected among the plantlets. The genetic integrity of in vitro grown plantlets was previously evaluated using the ISSR marker by

Kedra and Bach [51] in *L. martagon* and *L. longiflorum* Oriental hybrid 'Triumphator', *L. davidii* var. *unicolor*, Lilium Oriental hybrid 'Siberia', Lilium Asiatic hybrid 'Elite' and *L. formolongi* Bi et al. [52]. In agreement with the present study, in vitro regenerated plantlets through adventitious bud regeneration from scale explants in lilies can be considered genetically stable.

Cytokinins play a significant role in various physiological aspects of plant tissue culture, including promoting shoot formation, enhancing the quality of explants obtained from in vitro culture, influencing plant cell division, differentiation, chlorophyll accumulation, and delaying leaf senescence. Notably, among cytokinins, BA is the most utilized in micropropagation techniques. It is highly effective for in vitro shoot propagation, widely employed in commercial laboratories, and more cost-effective than naturally occurring cytokinins [53, 54]. However, the use of BA to induce shoot production in woody and herbaceous species can lead to challenges such as hyperhydricity, shoot-tip necrosis, inadequate rooting, and reduced survival rates [55, 56]. Moreover, high concentrations of BA have often been implicated in generating genetic diversity among regenerated plantlets [57]. The potential in vitro usage of mT as an alternative to BA has been evaluated in many species since its discovery and the initial research published concerning the metabolism and in vitro impacts of mT in micropropagated *Spathiphyllum floribundum* [58] It is shown that mT improved multiplication rate, rooting, quality of shoot and survival ability of in vitro grown plants [59]. In this study, the effects of mT are evaluated for the first time. Different concentrations of mT can be used alone or combined with the low level of auxin for in vitro conservation or micropropagation of lilies.

According to our results of ML application, in conjunction with the literature presented by Kirkis et al. [36], Jafari et al. [60], Aasim et al. [61, 62], and Demirel et al. [63], collectively shed light on the diverse applications of machine learning (ML) algorithms in the realm of plant tissue culture and micropropagation. Each study brings unique insights into optimizing growth

conditions, predicting outcomes, and enhancing the efficiency of in vitro regeneration protocols.

In our study, we employed XGBoost and MLP algorithms to analyze data related to the adventitious induction of buds and shoot proliferation in Lilium akkusianum. The performance metrics, including R2, Mean Absolute Error (MAE), and Root Mean Square Error (RMSE), provided a comprehensive evaluation. Notably, XGBoost outperformed MLP in terms of competitive RMSE outcomes, lower MAE, and greater R2 values, particularly excelling in predicting Root Length across various plant characteristics.

The findings of Kirkis et al. [36] on the in vitro whole plant regeneration of desi chickpea underscore the importance of ML algorithms in predicting shoot count and length. The study showcases the effectiveness of Random Forest (RF) in achieving high performance, as indicated by R2 values of 0.99 for shoot count and 0.98 for shoot length. The authors emphasize the utility of RF for precise predictions in tissue culture studies.

Jafari et al. [60] tackle the challenging aspect of in vitro adventitious rooting in Passiflora caerulea. The integration of a hybrid model, generalized regression neural network (GRNN) with genetic algorithm (GA), proves successful in predicting rooting responses. The study emphasizes the accuracy of the GRNN-GA model, showcasing its reliability in forecasting in vitro rooting outcomes. Aasim et al. [62] explore the use of ML algorithms in the germination of hemp seeds, highlighting the efficacy of the Random Forest model in predicting output variables with high F1 scores. The study demonstrates the potential of ML in optimizing conditions for seed germination and seedling growth. In a similar vein, Aasim et al. [61] delve into the in vitro regeneration of common beans, combining ML models with artificial neural networks. The Multilayer Perceptron (MLP) model emerges as the most effective in predicting and optimizing output variables, offering valuable insights for biotechnological applications in common bean breeding programs. Demirel et al. [63] explore the optimal conditions for the

growth of black chokeberry using tissue culture techniques, employing ML algorithms for predictive modeling. XGBoost and Support Vector Machine (SVM) models stand out in predicting various in vitro parameters, showcasing superior performance compared to other models.

Collectively, these studies underscore the versatility of ML algorithms in predicting, optimizing, and enhancing various aspects of plant tissue culture. The application of ML not only contributes to the efficiency of in vitro protocols but also offers a reliable means of forecasting outcomes, paving the way for precision agriculture and biotechnological advancements in plant sciences. The integration of ML into plant tissue culture studies holds immense potential for improving the success rates of micropropagation and addressing challenges in the field.

Conclusions

In conclusion, *L. akkusianum* R. Gämperle, an endemic Lilium species, successfully propagated from in vitro scale culture through in direct regeneration for the first time. Efficient callus induction and shoot proliferation were obtained. Meta-Topolin, compared to BA, increases adventitious bud formation per explants and shoot proliferation. IBA is more appropriate than NAA to improve rooting, root number, root length and survival rate of the in vitro grown plantlets.

Histological analysis confirmed that adventitious bud derived from callus tissues and ISSR markers proved the genetic stability of the plantlets. This protocol is highly efficient inducing callus and shoot regeneration and can potentially conserve the valuable germplasm of endemic *L. akkusianum* genotypes. Additionally, this optimized protocol enables in vitro or in vitro assisted studies, such as propagation via a bioreactor system, genetic transformation, or improving novel cultivars in L. akkusianum R. Gämperle.

The consistent and uninterrupted provision of robust plant material enabled by these refined protocols corresponds with the increasing need for *L. akkusianum* in diverse applications. As forthcoming investigations explore the synergy between machine learning (ML) and in vitro techniques, there exists the possibility of revealing innovative insights and strategies to enhance the sustainability and efficacy of plant propagation methods. The integration of machine learning into lily micropropagation signifies a forward-thinking approach in leveraging state-of-the-art technologies for the preservation and utilization of genetic resources in agricultural and conservation.

Acknowledgments

Thanks to Dr. Hakan Yilmaz, Department of Forestry, Akkuş Vocational School, Ordu University, for the botanical identification of *L. akkusianum*; Dr. Özhan Şimşek, Horticulture Department, Erciyes University for the technical support in molecular techniques; Dr. Şenay Karabıyık, Horticulture Department, Çukurova University, for the technical support in histological analysis.

References

[1] Ikinci, N.; Oberprieler, C.; Güner, A. On the origin of European lilies: phylogenetic analysis of Lilium section Liriotypus (Liliaceae) using sequences of the nuclear ribosomal transcribed spacers. Willdenowia 2006, 36(2), 647–656. https://doi.org/10.3372/wi.36.36201.

[2] Pelkonen, V.P.; Pirttila, A.M. Taxonomy and phylogeny of the genus Lilium. Floric. Ornam. Biotechnol. 2012, 6(2), 1–8.

[3] Uzunömeroğlu, H.Ü.Determination of morphological, anatomical, micromorphological and some biochemical properties of endemic Lilium akkusianum R. gamperle species

distributed in Ordu province. M.Sc. Thesis, Ordu Üniversitesi Fen Bilimleri Enstitüsü, Ordu, 2022.

[4] Özcan, Ö. Phenological and morphological characterization of Lilium candidum and Lilium akkusianum grown in Tokat ecological conditions, M.Sc. Thesis, Tokat Gaziosmanpaşa Üniversitesi, Fen Bilimleri Enstitüsü, Tokat. 2019.

[5] Ikinci, N.; Oberprieler, C. Genetic relationships among NE Turkish Lilium L.(Liliaceae) species based on a random amplified polymorphic DNA analysis. Plant Syst. Evol. 2010, 284(1), 41–48. https://doi.org/10.1007/s00606-009-0239-8.

[6] Demir, S.; Çelikel, F.G.; Endangered Lilium species of Turkey. Turk. J. Agric. F. Sci. Technol. 2017, 5(13), 1796–1801. https://doi.org/10.24925/turjaf.v5i13.1796-1801.1506.

[7] İzgü, T.; Sevindik, B.; Çürük, P.; Şimşek, Ö.; Aka Kaçar, Y.; Teixeira da Silva, J.A.; Yalçın Mendi, Y. Development of an efficient regeneration protocol for four Cyclamen species endemic to Turkey. Plant Cell, Tissue Organ Cult. 2016, 127(1), 95–113. https://doi.org/10.1007/s11240-016-1033-2.

[8] Şimşek, Ö.; Dönmez, D.; Sarıdaş, M.A.; Acar, E.; Kaçar, Y.A.; Kargı, S.P.; İzgü, T. In vitro and ex vitro propagation of Turkish myrtles through conventional and plantform bioreactor systems. PeerJ, 2023, 11, e16061. https://doi.org/10.7717/peerj.16061.

[9] Bahr, L.R.; Compton, M.E. Competence for in vitro bulblet regeneration among eight Lilium genotypes. HortScience 2004, 39(1), 127-129. https://doi.org/10.21273/HORTSCI.39.1.127.

[10] Varshney, A.; Dhawan, V.; Srivastava, P.S. A protocol for in vitro mass propagation of Asiatic hybrids of lily through liquid stationary culture. In Vitro Cell. Dev. Biol. Plant. 2000, 36(5), 383–391. https://doi.org/10.1007/s11627-000-0069-4.

[11] Youssef, N.M.; Shaaban, S.A.; Ghareeb, Z.F.; Taha, L.S.; In vitro bulb formation of direct and indirect regeneration of Lilium orientalis cv.“Starfighter” plants. Bull. Natl. Res. Cent. 2019, 43(1), 1–9. https://doi.org/10.1186/s42269-019-0246-z.

[12] Bakhshaie, M.; Khosravi, S.; Azadi, P.; Bagheri, H.; van Tuyl, J.M. Biotechnological advances in Lilium. Plant Cell Reports 2016, 35, 1799-1826. https://doi.org/10.1007/s00299-016-2017-8.

[13] Jin, S.; Wang, J.; Wang, X.; Sun, D.; Li, G.; Genovesi, A.D.; Liu, S. Direct and indirect shoot and bulblet regeneration from cultured leaf explants of Lilium pumilum, an endangered species. In vitro Cell Dev. Biol. Plant. 2014, 50:69–75. https://doi.org/10.1007/s11627-013-9573-1.

[14] Rafiq, S; Rather, Z.A.; Bhat, R.A.; Nazki, I.T.; Al-Harbi, M.S.; Banday, N.; Farooq, I.; Samra, B.N.; Khan, M.H.; Ahmed, A.F.; Andrabi, N. Standardization of in vitro micropropagation procedure of Oriental Lilium Hybrid Cv.‘Ravenna’. Saudi Journal of Biological Sciences 2021, 28(12), 7581-7587. https://doi.org/10.1016/j.sjbs.2021.09.064.

[15] Azadi, P.; Khosh-Khui, M. Micropropagation of Lilium ledebourii (Baker) Boiss as affected by plant growth regulator, sucrose concentration, harvesting season and cold treatments. Electronic Journal of Biotechnology 2007, 10(4), 582-591. http://dx.doi.org/10.4067/S0717-34582007000400011.

[16] Liu, J.; Zhang, J.; Xu, B.; Jia, C.; Zhang, J.; Tan, G.; Jin, Z. Regeneration and production of transgenic Lilium longiflorum via Agrobacterium tumefaciens. In vitro Cell Dev. Biol. Plant 2011, 47, 348–356. https://doi.org/10.1007/s11627-010-9336-1.

[17] Tang, Y.P.; Liu, X.Q.; Gituru, R.W.; Chen, L.Q.. Callus induction and plant regeneration from in vitro cultured leaves, petioles and scales of Lilium leucanthum (Baker) Baker. Biotechnology & Biotechnological Equipment 2014, 24(4), 2071-2076. https://doi.org/10.2478/V10133-010-0077-4.

[18] Azeri, F.N.,Öztürk, G. Microbulb and plantlet formation of a native bulbous flower, Lilium monodelphum M. Bieb, var. Armenum, through tissue culture propagation. Biotechnology Reports 2021, 32, e00665. https://doi.org/10.1016/j.btre.2021.e00665.

[19] Patil, A.M.; Gunjal, P.P.; Das, S. In vitro micropropagation of Lilium candidum bulb by application of multiple hormone concentrations using plant tissue culture technique. International Journal for Research in Applied Sciences and Biotechnology 2021, 8(2), 244-253. https://doi.org/10.3103/ijrasb.8.2.32.

[20] Skorić, M.; Živković, S.; Savić, J.; Šiler, B.; Sabovljević, A.; Todorović, S.; Grubišić, D. Efficient one-step tissue culture protocol for propagation of endemic plant, Lilium martagon var. cattaniae Vis. African Journal of Biotechnology, 2012, 11(8), 1862-1867. https://doi.org/10.5897/AJB11.2076.

[21] Nhut, D.T.; Van L.B.; Tanaka, M.; Van K.T.T. Shoot induction and plant regeneration from receptacle tissues of Lilium longiflorum. Scientia Horticulturae, 2001, 87(1-2), 131-138. https://doi.org/10.1016/S0304-4238(00)00163-1.

[22] Gentile, A.; Frattarelli, A.; Nota, P.; Condello, E.; Caboni, E. The aromatic cytokinin meta-topolin promotes in vitro propagation, shoot quality and micrografting in Corylus colurna L. Plant Cell, Tissue and Organ Culture 2017, 128, 693-703. https://doi.org/10.1007/s11240-016-1150-y.

[23] Doležal, K.; Bryksová, M. Topolin metabolism and its implications for in vitro plant micropropagation. In Meta-topolin: A Growth Regulator for Plant Biotechnology and Agriculture, 1st ed., Ahmad, N.; Strnad, M., Eds.; Springer: Singapore, 2021, pp. 49-58. https://doi.org/10.1007/978-981-15-9046-7_6.

[24] Werbrouck, S. P. (2021). Meta-topolin and related cytokinins as a solution to some in vitro problems. In Meta-topolin: A Growth Regulator for Plant Biotechnology and Agriculture,

1st ed., Ahmad, N.; Strnad, M., Eds.; Springer: Singapore, pp. 85-91. https://doi.org/10.1007/978-981-15-9046-7_9.

[25] Nowakowska, K.; Pacholczak, A. (Comparison of the effect of meta-topolin and benzyladenine during Daphne mezereum L. micropropagation. Agronomy, 2020, 10(12), 1994. https://doi.org/10.3390/agronomy10121994.

[26] Mahanta, M.; Gantait, S.; Mukherjee, E.; Bhattacharyya, S. meta-Topolin-induced mass propagation, acclimatization and cyto-genetic fidelity assessment of gerbera (Gerbera jamesonii Bolus ex Hooker f.). South African Journal of Botany, 2023, 153, 236-245. https://doi.org/10.1016/j.sajb.2022.11.032.

[27] Verma, V.; Kumar, A.; Priti, S.; Thakur, M.; Bhargava, B. Meta-Topolin mediated in vitro propagation in an ornamentally important crop Iris× hollandica Tub. cv. professor Blaauw and genetic fidelity studies using SCoT markers. Plant Cell, Tissue and Organ Culture (PCTOC), 2022, 151(3), 681-694. https://doi.org/10.1007/s11240-022-02395-1.

[28] Sochacki, D.; Marciniak, P.; Ciesielska, M.; Zaród, J.; Sutrisno. The influence of selected plant growth regulators and carbohydrates on in vitro shoot multiplication and bulbing of the Tulip (Tulipa L.). Plants 2023, 12(5), 1134. https://doi.org/10.3390/plants12051134.

[29] Meyer, E.M.; Touchell, D.H.; Ranney, T.G. In vitro shoot regeneration and polyploid induction from leaves of Hypericum species. HortScience, 2009, 44(7), 1957-1961. https://doi.org/10.21273/HORTSCI.44.7.1957.

[30] Luo, Y.; Zheng, K.; Liu, X.; Tao, J.; Sun, X.; Deng, Y.; Deng, X. In vitro propagation and genetic uniformity assessment of Manglietiastrum sinicum: A critically endangered Magnoliaceae species. Plants 2023, 12(13), 2500. https://doi.org/10.3390/plants12132500.

[31] Xi, M.; Sun, L.; Qiu, S.; Liu, J.; Xu, J.; Shi, J. In vitro mutagenesis and identification of mutants via ISSR in lily (Lilium longiflorum). Plant Cell Reports 2012, 31, 1043-1051. https://doi.org/10.1007/s00299-011-1222-8.

[32] Yin, Z.F.; Zhao, B.; Bi, W.L.; Chen, L.; Wang, Q.C. Direct shoot regeneration from basal leaf segments of Lilium and assessment of genetic stability in regenerants by ISSR and AFLP markers. In Vitro Cellular & Developmental Biology-Plant 2013, 49, 333-342. https://doi.org/10.1007/s11627-013-9501-4.

[33] Yang, L.; Chao, L.; Su, X.; Wang, C.; Dong, C.; Chen, S. High-frequency in vitro plantlet regeneration in Lilium davidii var. unicolour Salisb, an important edible and medicinal plant, and confirmation of genetic fidelity of regeneration plantlets using ISSR markers. Plant Biotechnology Reports, 2021, 15(4), 435-446. https://doi.org/10.1007/s11816-021-00690-5.

[34] Hesami, M.; Jones, A.M.P. Application of artificial intelligence models and optimization algorithms in plant cell and tissue culture. Appl. Microbiol. Biotechnol. 2020, 104, 9449-9485. https://doi.org/10.1007/s00253-020-10888-2

[35] Hesami, M.; Pepe, M.; Monthony, A.S.; Baiton, A.; Jones, A.M.P. Modeling and optimizing in vitro seed germination of industrial hemp (Cannabis sativa L.). Ind Crops Prod. 2021, 170, p.113753. https://doi.org/10.1016/j.indcrop.2021.113753

[36] Kirtis, A.; Aasim, M.; Katırcı, R. Application of artificial neural network and machine learning algorithms for modeling the in vitro regeneration of chickpea (Cicer arietinum L.). Plant Cell, Tissue Organ Cult. 2022, 150(1), 141-152. https://doi.org/10.1007/s11240-022-02255-y

[37] Emamgholizadeh, S., Parsaeian, M., & Baradaran, M. Seed yield prediction of sesame using artificial neural network. European Journal of Agronomy 2015, 68, 89-96. https://doi.org/10.1016/j.eja.2015.04.010

[38] Murashige, T.; Skoog, F.A revised medium for rapid growth and bio assays with tobacco tissue cultures. Physiol. Plant. 1962, 15, 473–496. https://doi.org/10.1111/j.1399-3054.1962.tb08052.x.

[39] Karabiyik, S.; Eti, S. Determination of flowering dates and pollen properties of some pecan nut cultivars in Adana ecological conditions. Turk. J. Agric. F. Sci. Technol. 2018, 6(12), 1795–1801. https://doi.org/10.24925/turjaf.v6i12.1795-1801.2112.

[40] Karabiyik, S.; Eti, S. Nucellar embryony and formation mechanism in sour orange. YYU J. Agric. Sci. 2020, 30(4), 761–771. https://doi.org/10.29133/yyutbd.741804.

[41] Edwards, K; Johnstone, C.; Thompson, C. A simple and rapid method for the preparation of plant genomic DNA for PCR analysis. Nucleic Acids Res. 1991, 19(6), 1349. https://doi.org/10.1093/nar/19.6.1349.

[42] Genişel, H. Characterization of new putative species crocus (Colchicum L.) in Turkey's Flora using ISSR markers. M.Sc. Thesis, University of Istanbul, Institute of Science, 2013.

[43] Bakhshaie, M.; Babalar, M.; Mirmasoumi, M.; Khalighi, A. Somatic embryogenesis and plant regeneration of Lilium ledebourii (Baker) Boiss., an endangered species. Plant Cell, Tissue Organ Cult. 2010, 102, 229–235. https://doi.org/10.1007/s11240-010-9726-4.

[44] Fu, L.; Zhu, Y.; Li, M.; Wang, C.; Sun, H. Autopolyploid induction via somatic embryogenesis in Lilium distichum Nakai and Lilium cernuum Komar. Plant Cell, Tissue Organ Cult. 2019, 139, 237–248. https://doi.org/10.1007/s11240-019-01671-x.

[45] Nam, S.W.; Kim, H.Y. Plant regeneration through adventitious bud formation and callus induction from scales of Lilium lancifolium Thunb. Journal of Plant Biotechnology, 2003, 30(1), 53-58. https://doi.org/10.5010/JPB.2003.30.1.053.

[46] Lopez-Carbonell, M.; Alegre, L.; Prinsen, E.; Van Onckelen, H. Diurnal fluctuations of endogenous IAA content in aralia leaves. Biologia Plantarum 1992, 34(3-4), 223-227. https://doi.org/10.1007/BF02925873.

[47] Joshi, H.; Nekkala, S.; Soner, D.; Kher, M.M.; Nataraj, M. In vitro shoot multiplication of Withania coagulans (stocks) Dunal. Plant Tissue Culture and Biotechnology 2016, 26(2), 187-195.

[48] Liu, X.; Yang, G. Adventitious shoot regeneration of oriental lily (Lilium orientalis) and genetic stability evaluation based on ISSR marker variation. In Vitro Cellular & Developmental Biology-Plant 2012, 48, 172-179. https://doi.org/10.1007/s11627-012-9429-0.

[49] Mir, J.I.; Ahmed, N.; Itoo, H.; Sheikh, M.A.; Rashid, R.; Wani, S.H. In vitro propagation of Lilium (Lilium longiflorum). Indian Journal of Agricultural Sciences 2012, 82(5), 455.

[50] Wawrosch, C.; Malla, P.R.; Kopp, B. Clonal propagation of Lilium nepalense D. Don, a threatened medicinal plant of Nepal. Plant Cell Reports 2001, 20, 285-288. https://doi.org/10.1007/s002990100322.

[51] Kedra, M.; Bach, A. Morphogenesis of Lilium martagon L. explants in callus culture. Acta Biologica Cracoviensia. Series Botanica, 2005, 47(1).

[52] Bi, W.L.; Chen, L.; Guo, L.; Pan, C.; Yin, Z.F.; Wang, Q.C. Plant regeneration via embryo-like structures: histological observations and genetic stability in regenerants of Lilium spp. J. Hortic. Sci. Biotechnol. 2015, 90(6), 626–634. https://doi.org/10.1080/14620316.2015.11668725.

[53] Bairu, M.W.; Stirk, W.A.; Dolezal, K.; Van Staden, J. Optimizing the micropropagation protocol for the endangered Aloe polyphylla: can meta-topolin and its derivatives serve as replacement for benzyladenine and zeatin?. Plant Cell, Tissue and Organ Culture 2007, 90, 15-23. https://doi.org/10.1007/s11240-007-9233-4.

[54] Koç, E. Effects of meta-topolin on the growth, physiological and biochemical parameters in plant tissue culture. In Meta-topolin: A Growth Regulator for Plant Biotechnology and Agriculture, 1st ed., Ahmad, N.; Strnad, M., Eds.; Springer: Singapore, 265-278, 2021. https://doi.org/10.1007/978-981-15-9046-7_19.

[55] Bairu, M.W.; Jain, N.; Stirk, W.A.; Doležal, K.; Van Staden, J. Solving the problem of shoot-tip necrosis in Harpagophytum procumbens by changing the cytokinin types, calcium and

boron concentrations in the medium. South African Journal of Botany 2009, 75(1), 122-127. https://doi.org/10.1016/j.sajb.2008.08.006.

[56] Amoo, S.O.; Finnie, J.F.; Van Staden, J. The role of meta-topolins in alleviating micropropagation problems. Plant Growth Regulation 2011, 63, 197-206. https://doi.org/10.1007/s10725-010-9504-7.

[57] Krishna, H.; Alizadeh, M.; Singh, D.; Singh, U.; Chauhan, N.; Eftekhari, M.; Sadh, R.K. Somaclonal variations and their applications in horticultural crops improvement. 3 Biotech 2016, 6, 1-18. https://doi.org/10.1007/s13205-016-0389-7.

[58] Werbrouck, S. P., Strnad, M., Van Onckelen, H. A., & Debergh, P. C. Meta□topolin, an alternative to benzyladenine in tissue culture?. Physiologia plantarum 1996, 98, 2, 291-297. https://doi.org/10.1034/j.1399-3054.1996.980210.x.

[59] Zaytseva, Y. G., Ambros, E. V., Novikova, T. I. Meta-topolin: advantages and disadvantages for in vitro propagation. Meta-topolin: a growth regulator for plant biotechnology and agriculture. In Meta-topolin: A Growth Regulator for Plant Biotechnology and Agriculture, 1st ed., Ahmad, N.; Strnad, M., Eds.; Springer: Singapore, 119-141, 2021. https://doi.org/10.1007/978-981-15-9046-7_11

[60] Jafari, M.; Daneshvar, M.H.; Jafari, S.; Hesami, M. Machine learning-assisted in vitro rooting optimization in passiflora caerulea. Forests 2022, 13(12), 2020. https://doi.org/10.3390/f13122020

[61] Aasim, M., Katirci, R., Baloch, F. S., Mustafa, Z., Bakhsh, A., Nadeem, M. A., ... & Chung, Y. S. Innovation in the breeding of common bean through a combined approach of in vitro regeneration and machine learning algorithms. Frontiers in Genetics 2022, 13, 897696. https://doi.org/10.3389/fgene.2022.897696

[62] Aasim, M., Katırcı, R., Akgur, O., Yildirim, B., Mustafa, Z., Nadeem, M. A., ... & Yılmaz, G. Machine learning (ML) algorithms and artificial neural network for optimizing in

vitro germination and growth indices of industrial hemp (Cannabis sativa L.). Industrial Crops and Products 2022, 181, 114801.

[63] Demirel, F.; Uğur, R.; Popescu, G.C.; Demirel, S.; Popescu, M. Usage of Machine Learning Algorithms for Establishing an Effective Protocol for the In Vitro Micropropagation Ability of Black Chokeberry (Aronia melanocarpa (Michx.) Elliott). Horticulturae 2023, 9(10), 1112. https://doi.org/10.3390/horticulturae9101112

Click here to access/download;Figure;Figure 1.docx

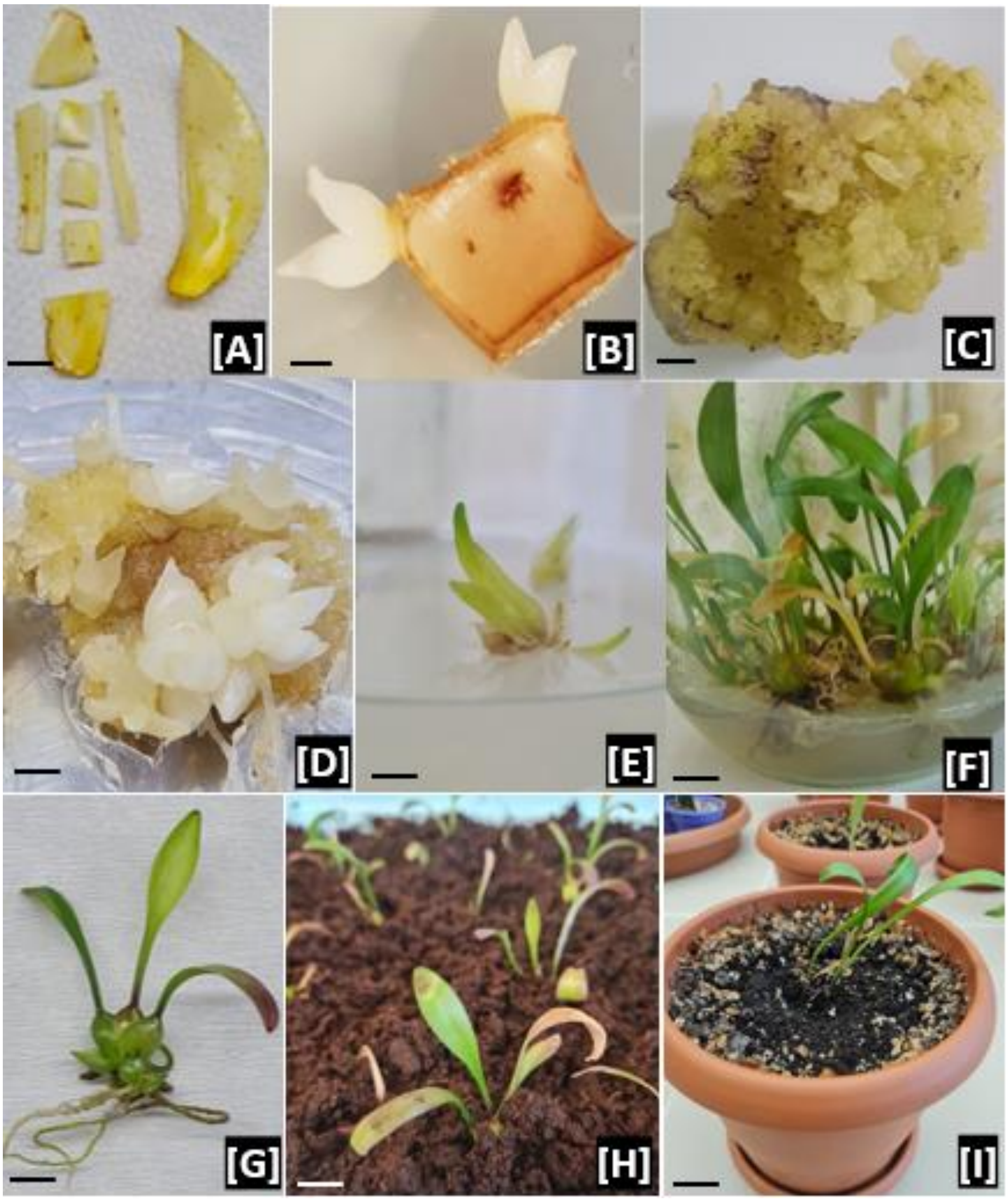

Figure 1. In vitro regeneration in *L. akkusianum*. (A) Explant preparation. (B) Adventitious bud regeneration in M0 medium 5-6 weeks of culture in the dark. (C) Callus and adventitious buds regeneration in M9 medium 5-6 weeks of culture. (D) Callus and adventitious buds regeneration in M10 medium 5-6 weeks of culture. (E) Shoot development in M12 medium 2 weeks after culture initiation under 16/8 h photoperiod. (F) Later shoots development and proliferation under 16/8 h photoperiod (G) Plantlets with well-developed roots. (H) Acclimatized plantlets in the grow box (I) Plantlets 2 months after transfer into the peat-perlite substrate (1:1 v/v). Scale bars: B, D, E, F = 10 mm; A, C, G, H = 1 cm; I =5 cm.

Figure 2 Click here to access/download;Figure;Figure 2.docx

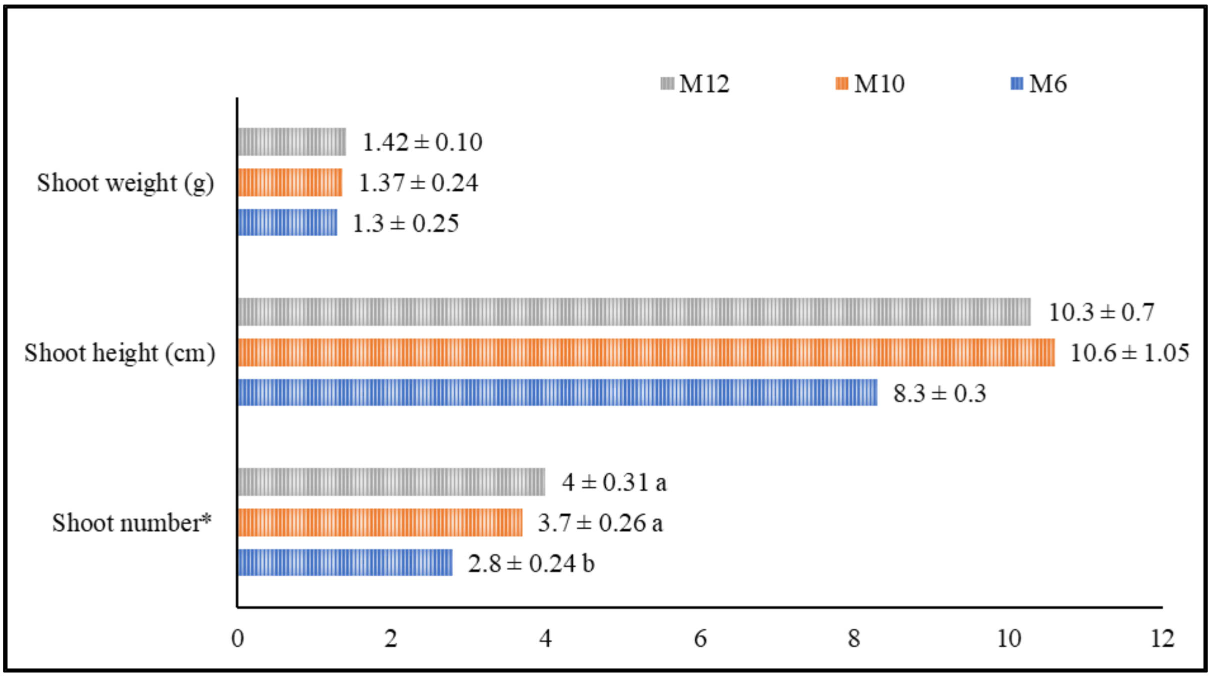

Figure 2. Effects of plant growth regulator combination on shoot number, fresh shoot height and shoot weight in the sixth week of culture (*$p < 0.05$, $LSD_{shootnumber} = 0.876$; Levels not connected by the same letter are significantly different).

Click here to access/download;Figure;Figure 3.docx

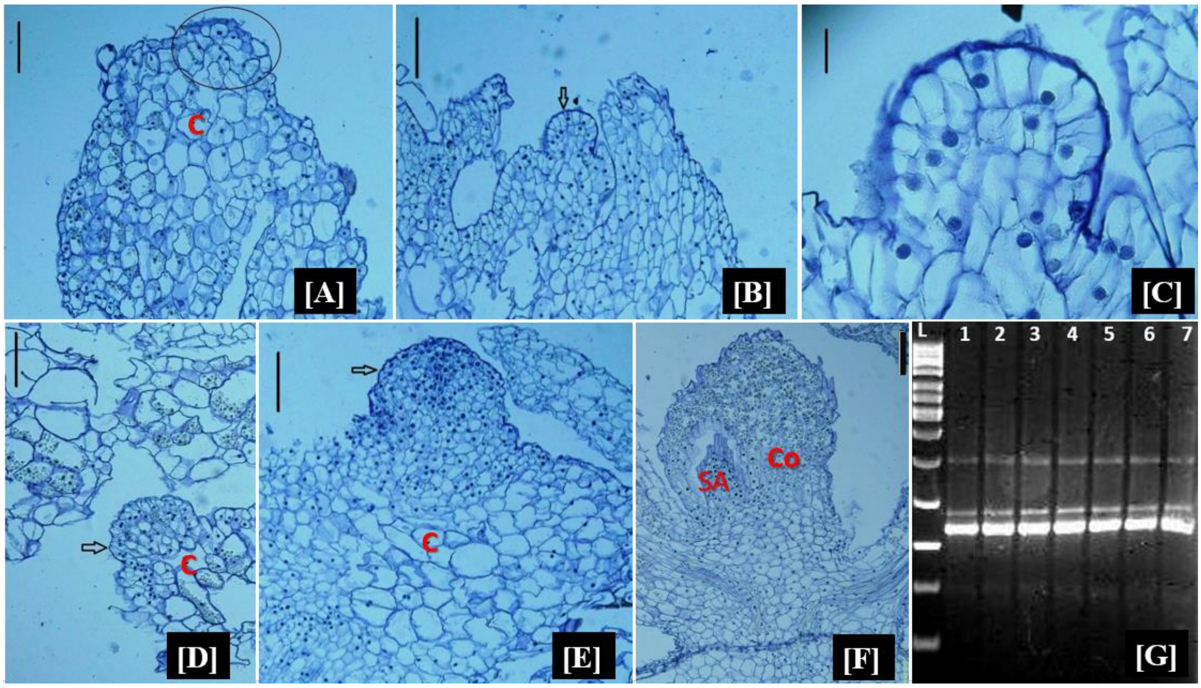

Figure 3. Histological observations on developmental stages of plant regeneration and genetic stability of in vitro regenerated plants. (A, B) General view of callus (C) and new cells. (C) Cell division and morphogenesis (D, E) Initiation of adventitious bud on callus tissue (black arrow). (E) Mature adventitious buds with shoot apical meristem (SA) and cotyledone tissues (Co), (G) DNA bands profile of in vitro regenerated plantlets (2-7) and mother plant (1). Scale bars: A, B, D, E and F: 100 µm, C: 20 µm

Figure 4 Click here to access/download;Figure;Figure 4.docx

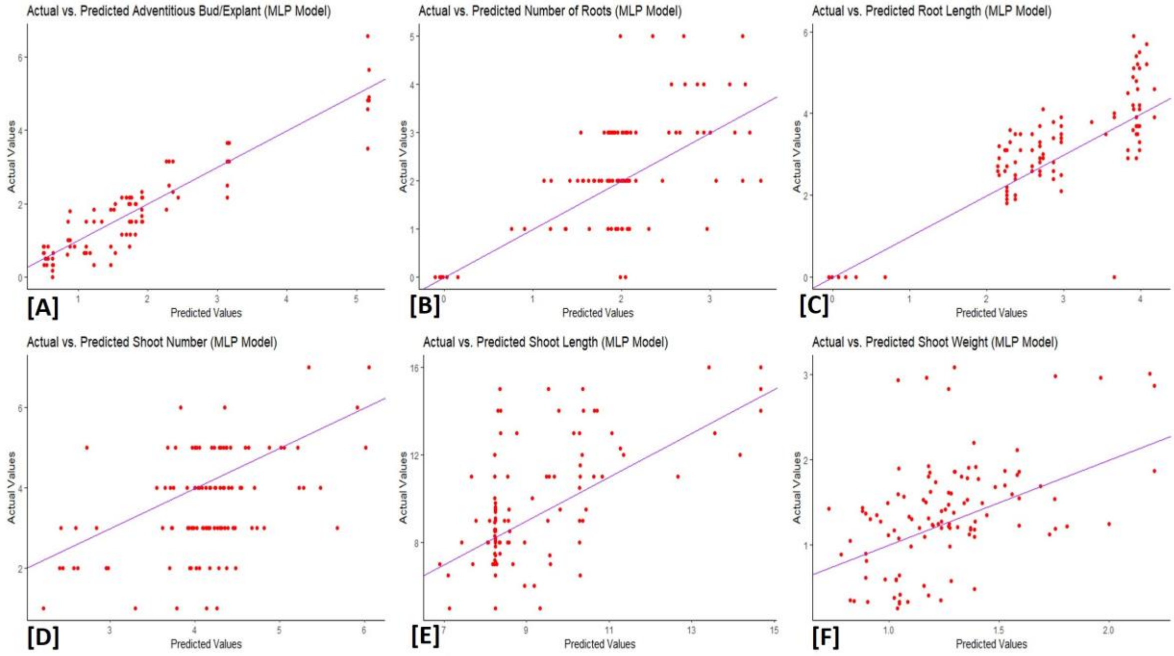

Figure 4. Actual against predicted values of (A) adventitious bud, (B) number of roots, (C) root length, (D) number of shoots, (E) shoot length (F) shoot weight using MLP model analysis.

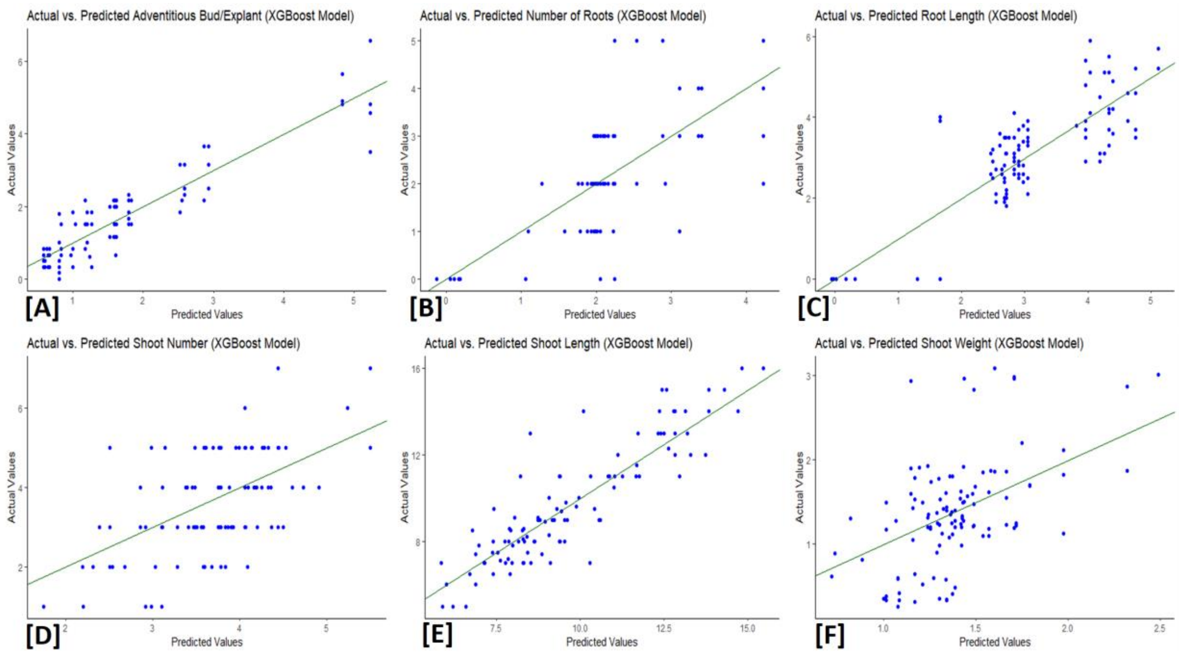

Figure 5. Actual against predicted values of (A) adventitious bud, (B) number of roots, (C) root length, (D) number of shoots, (E) shoot length (F) shoot weight using XGBoost model analysis.
