# Supplementary material for: Application of machine learning in in vitro propagation of endemic Lilium akkusianum R. Gämperle
Source: PLoS One. 2024 Jul 25;19(7):e0307823. doi: 10.1371/journal.pone.0307823 (PMC11271868; doi:10.1371/journal.pone.0307823)
Supplement: Attachment — Submitted filename: Response to Reviewers Letter.docx [file pone.0307823.s009.docx]

Response to Reviewers

**Reviewer 1**

**Comment:** Writing Quality: There are concerns about the clarity and quality of writing throughout the manuscript. It is recommended that you seek the assistance of an expert in scientific writing to improve the English language and presentation of your work.

**Response:** Thank you for your point out. The English language of revised manuscript edited by Birol Kurt who is professional language translator at Ondokuz Mayıs University, R&D Coordination Office, Academic Writing Advisory Unit.

**Comment:** Species Name Formatting: Please ensure that the species name, Lilium akkusianum, is presented in full and italicized upon its first mention in the abstract, and maintain correct formatting throughout the text.

**Response:** Thank you. All species name checked and revised throughout the text.

**Comment:** Introduction Section: The introduction should focus specifically on research progress related to Lilium akkusianum rather than a broad overview of tissue culture in different species. Any comparison with other species should be moved to the discussion section.

**Response:** Thank you for your valuable suggestion, but we don’t agree with you. This is the first report on the propagation of endemic lily species. Also, literature about the usage of meta-topolin in *in vitro* propagation in ornamental plants is very limited. Therefore, we believe that giving literature rather than *L. akkusianum* species but close to current studies in introduction part will provide a background to readers to understand why this study was conducted clearly.

**Comment:** Histological Sections: The relevance and quality of the histological sections presented are questioned. It is advised to remove these sections, except for Figure 3G, which was deemed valuable.

**Response:** Thank you for your contributions and advice. Histological section removed from the manuscript, and Figure 3G integrated with Figure 1.

**Comment:** Formatting: Ensure that all instances of Latin names and terms such as in vitro/ex vitro are italicized throughout the manuscript.

**Response:** Thank you. All Latin names checked and italicized throughout the text.

**Comment:** Machine learning is not a micropropagation technique. It is an analysis method used in micropropagation research. The reports confirm the reliability and accuracy of artificial intelligence in micropropagation systems. On the other hand, they are methods of analyzing data. I mean that micropropagation techniques are meristem culture, embryo culture, callus culture e.t.c. Additionally the manuscript presents a valuable, conventional, micropropagation method by scales and various phytohormones on solid MS media. To my opinion, there have not been used advanced micropropagation techniques and the title should be revised. It is about an application of ML in in vitro propagation of endemic lily Lilium akkusianum R. Gämperle.

The title must be focused on in vitro propagation and the usage/application of ML.

**Comment:** Thank you very much for your valuable comments. I have revised the title as “Application of machine learning in in vitro propagation of endemic Lilium akkusianum R. Gämperle”

Reviewer #2:

**Comment:** General Comments The study presents a suitable micropropagation method for Lilium akkusiamum, using various phytohormones. This is the first study on this endemic species. The genetic stability of regenerated plantlets was assessed as well by molecular markers. Machine learning methods were evaluated as well. The reference list is extended with recent manuscripts. There are no unnecessary references. A general comment is that in vitro/ex vitro etc and scientific names must be written in italics everywhere in the text.

**Response:** Thank you very much for your efforts and valuable contributions to study. All scientific names checked and rewritten in italics.

**Introduction**

**Comment:** The background is OK. There are information relevant studies and in vitro methods in the genus Lilium. The aim is clearly presented.

**Response:** Thank you for your comment.

**Comment:** Material and Methods Well and clear presented. Statistical methods, design and replications suitable for analysis.

**Comment:** Results: Well presented, including the necessary figures, tables and pictures.

Discussion: Well presented. It contains the necessary references.

There are some issues in the attached .pdf

To my opinion there are two points to be revised:

1. Title

“Integrating machine learning in endemic lily conservation: advanced micropropagation techniques for Lilium akkusianum R. Gämperle”

Comment:

Machine learning is not a micropropagation technique. It is an analysis method used in micropropagation research. The reports confirm the reliability and accuracy of artificial intelligence in micropropagation systems. On the other hand, they are methods of analyzing data. I mean that micropropagation techniques are meristem culture, embryo culture, callus culture e.t.c. Additionally the manuscript presents a valuable, conventional, micropropagation method by scales and various phytohormones on solid MS media. To my opinion, there have not been used advanced micropropagation techniques and the title should be revised. It is about an application of ML in in vitro propagation of endemic lily Lilium akkusianum R. Gämperle.

The title must be focused on in vitro propagation and the usage/application of ML.

**Response:** Thank your vey much your consideration. I agree that the revision of title, so title revised as “Application of machine learning in in vitro propagation of endemic *Lilium akkusianum* R. Gämperle”

2. Conclusions

Comment:

I think that they need revision. There are general comments on the use of ML. Did a suitable ML-method revealed?

**Response:** Thank you very much for your point out this. It is stated in the discussion section which model gives better results. However, it was missing in conclusion part as you stated. Therefore, we stated it in conclusion part as; “In this study, XGBoost and MLP algorithms were employed to model and predict adventitious bud induction, shoot proliferation, and rooting from scale explants of *L. akkusianum*. XGBoost outperformed MLP in terms of competitive *RMSE* outcomes, lower *MAE*, and greater *R^2^* values which means it is a more suitable model than MLP for micropropagation of *L. akkusianum*.”
