# Supplementary material for: Application of machine learning in in vitro propagation of endemic Lilium akkusianum R. Gämperle
Source: PLoS One. 2024 Jul 25;19(7):e0307823. doi: 10.1371/journal.pone.0307823 (PMC11271868; doi:10.1371/journal.pone.0307823)
Supplement: Attachment — Submitted filename: Response to Reviewers.docx [file pone.0307823.s011.docx]

**Response to Reviewers**

**Comment:** Reviewer #1: After the author can revised the following mini errors in the text, the manuscript can be accepted.

Line 199 µ?

Line 199, subscript;

The second paragraph or others need to be preceded by a few spaces；

Between two numbers in the text, use “‒”;

Conclusion section, it's too long, reducing the numbers and condensing the content.

**Response:** Thank you very much your valuable contribution. Mini errors in the text corrected. Units given in Line 199 re-checked and both are correct, therefore they were not changed.

We left spaces between paragraphs and used “‒” between two numbers in the text. Conclusion section revised and shortened.

**Journal Requirements:**

Please review your reference list to ensure that it is complete and correct. If you have cited papers that have been retracted, please include the rationale for doing so in the manuscript text, or remove these references and replace them with relevant current references. Any changes to the reference list should be mentioned in the rebuttal letter that accompanies your revised manuscript. If you need to cite a retracted article, indicate the article’s retracted status in the References list and also include a citation and full reference for the retraction notice.

**Response:**

We have reviewed the references and no retracted papers cited in the text. However, we noticed that there are two updated publication that are [11] and [27]. In the [11] publication, family name of correspondence was updated and in the [27] publication, Figure 2-3 captions and headings of Table 2-3 updated. Therefore, they haven't removed from the text.

References [15] and [19] are available on the journals website, but doi number of the papers doesn’t work, so doi numbers deleted from the reference list.

In the [45], doi number of the reference corrected.

[11]	Youssef, N.M.; Shaaban, S.A.; Ghareeb, Z.F.; Taha, L.S. In vitro bulb formation of direct and indirect regeneration of Lilium orientalis cv.“Starfighter” plants. Bull. Natl. Res. Cent. 2019, 43(1), 1–9. <https://doi.org/10.1186/s42269-019-0246-z>.

[15]	Azadi, P.; Khosh-Khui, M. Micropropagation of Lilium ledebourii (Baker) Boiss as affected by plant growth regulator, sucrose concentration, harvesting season and cold treatments. Electronic Journal of Biotechnology 2007, 10(4), 582-591.

[19]	Patil, A.M.; Gunjal, P.P.; Das, S. In vitro micropropagation of Lilium candidum bulb by application of multiple hormone concentrations using plant tissue culture technique. International Journal for Research in Applied Sciences and Biotechnology 2021, 8(2), 244-253.

[27]	Verma, V.; Kumar, A.; Priti, S.; Thakur, M.; Bhargava, B. Meta-Topolin mediated in vitro propagation in an ornamentally important crop Iris× hollandica Tub. cv. professor Blaauw and genetic fidelity studies using SCoT markers. Plant Cell, Tissue and Organ Culture (PCTOC), 2022, 151(3), 681-694 <https://doi.org/10.1007/s11240-022-02383-5>.

[45]	Joshi, H.; Nekkala, S.; Soner, D.; Kher, M.M.; Nataraj, M. In vitro shoot multiplication of Withania coagulans (stocks) Dunal. Plant Tissue Culture and Biotechnology 2016, 26(2), 187-195. https://doi.org/10.3329/ptcb.v26i2.30569.
